# Edible Crickets (Orthoptera) Around the World: Distribution, Nutritional Value, and Other Benefits—A Review

**DOI:** 10.3389/fnut.2020.537915

**Published:** 2021-01-12

**Authors:** Henlay J. O. Magara, Saliou Niassy, Monica A. Ayieko, Mukundi Mukundamago, James P. Egonyu, Chrysantus M. Tanga, Emily K. Kimathi, Jackton O. Ongere, Komi K. M. Fiaboe, Sylvain Hugel, Mary A. Orinda, Nanna Roos, Sunday Ekesi

**Affiliations:** ^1^School of Agricultural and Food Sciences, Jaramogi Oginga Odinga University Science and Technology (JOOUST), Bondo, Kenya; ^2^International Center of Insect Physiology and Ecology (icipe), Nairobi, Kenya; ^3^The International Institute of Tropical Agriculture (IITA), Yaoundé, Cameroon; ^4^Institut des Neurosciences Cellulaires et Intégratives, UPR 3212 CNRS-Université de Strasbourg, Strasbourg, France; ^5^Department of Nutrition, Exercise and Sports, University of Copenhagen, Frederiksberg, Denmark

**Keywords:** edible crickets, food, food security, distribution, nutritional value, medicine, cultural entomology

## Abstract

Edible crickets are among the praised insects that are gaining recognition as human food and livestock feed with a potential of contributing to food security and reduction of malnutrition. Globally, the sustainable use of crickets as food or feed is undermined by lack of information on the number of the edible crickets, the country where they are consumed, and the developmental stages consumed. Furthermore, lack of data on their nutritional content and the potential risks to potential consumers limits their consumption or inclusion into other food sources. We reviewed published literature on edible cricket species, countries where they are consumed, and the stage at which they are consumed. We further reviewed information on their nutritional content, the safety of cricket consumption, and the sensory qualities of the edible crickets. We also looked at other benefits derived from the crickets, which include ethnomedicine, livestock feed, pest management strategies, contribution to economic development, and livelihood improvement, particularly in terms of use as food preservatives and use within music, sports, and cultural entomology. Lastly, we reviewed information on the farming of edible crickets. In this review, we report over 60 cricket species that are consumed in 49 countries globally. Nutritionally, crickets are reported to be rich in proteins, ranging from 55 to 73%, and lipids, which range from 4.30 to 33.44% of dry matter. The reported amount of polyunsaturated fatty acids (PUFA) is 58% of the total fatty acids. Edible crickets contain an appreciable amount of macro- and micro-mineral elements such as calcium, potassium, magnesium, phosphorus, sodium, iron, zinc, manganese, and copper. Also, the crickets are rich in the required amount of vitamins such as B group vitamins and vitamins A, C, D, E, and K. Overall, the cricket species examined in this review are safe to be consumed, and they display high proximate content that can replace plant and livestock products. The crickets play valuable roles in contributing to the economies of many countries and livelihoods, and they have medicinal and social benefits. This review is expected to promote greater recognition of crickets as a source of food, feed, and other benefits in the world and encourage up-scaling by farming them for sustainable utilization.

## Introduction

**Figure 1:**
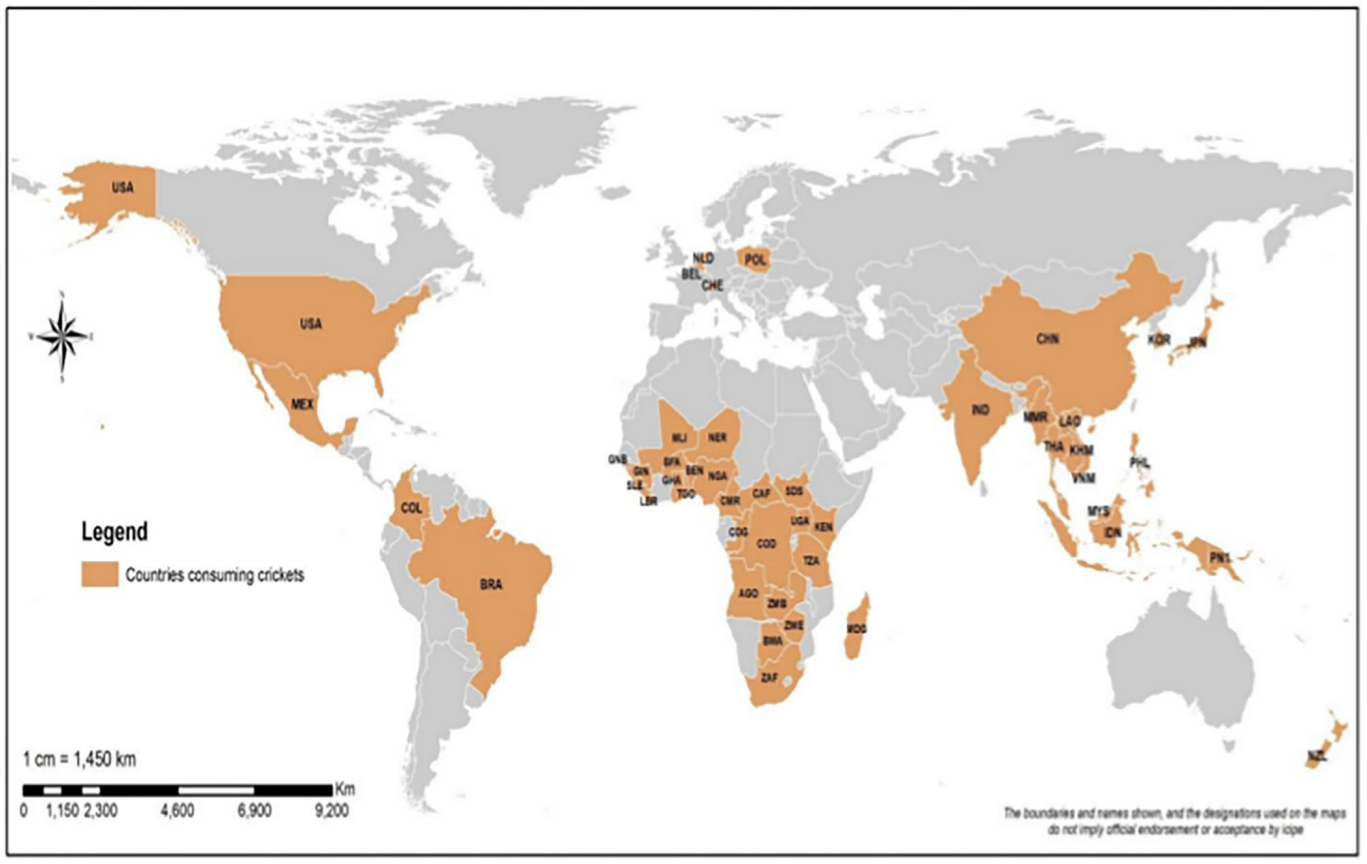
A world map showing countries where crickets are consumed as food. USA (United State of America), Mex (Mexico), Col (Columbia), Bra (Brazil), KEN (Kenya), ZMB (Zambia), GNB (Guinea Bissau), SLE (Sierre Leone), GIN (Guinée), LBR (Liberia), BEN (Benin), TGO (Togo), NGA (Nigeria), COD (Democratic Republic of Congo), SDS (South Sudan), UGA (Uganda), ZWE (Zimbabwe), TZA (Tanzania), NER (Niger), AGO (Angola), COG (Congo/Congo Brazzaville), BWA (Botswana), ZAF (South Africa), MLI (Mali), GHA (Ghana), CAF (Central African Republic), BFA (Burkina Faso), CMR (Cameroon), MDG (Madagascar), PNG (Papua New Guinea), NZL (New Zealand), NLD (Netherlands), BEL (Belgium), CHE (Switzerland), POL (Poland), THA (Thailand), PHL (Philippines), VNM (Viet Nam), IND (India), IDN (Indonesia), LAO (Laos People's Democratic Republic), KOR (South Korea), KHM (Cambodia), MYS (Malaysia), JPN (Japan), PNI (Sabah), MMR (Myanmar), CHN (China).

**Figure 2:**
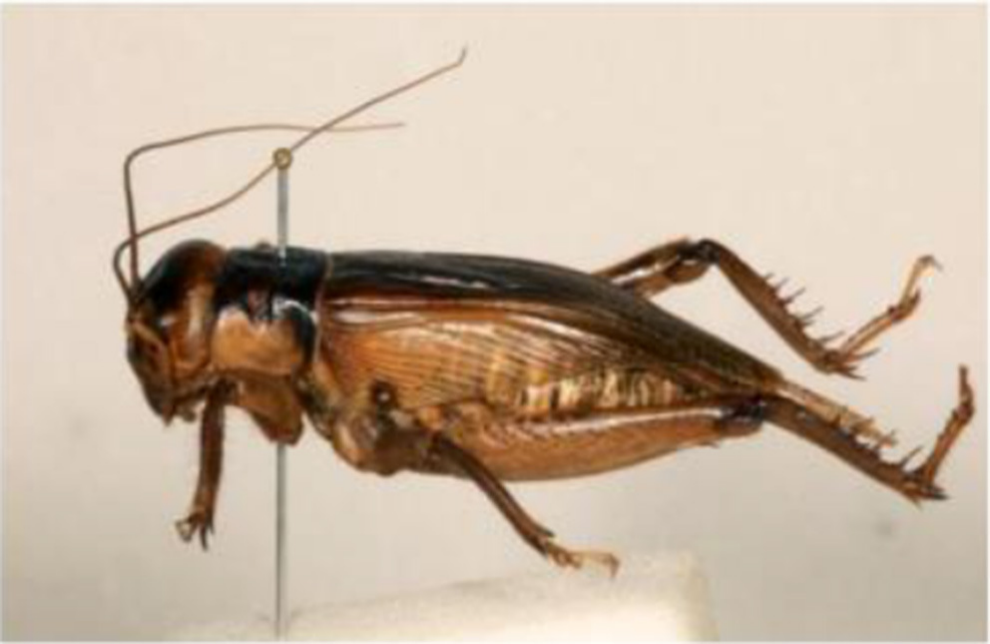
*Brachytrupes membranaceus*. Source: Authors.

**Figure 3:**
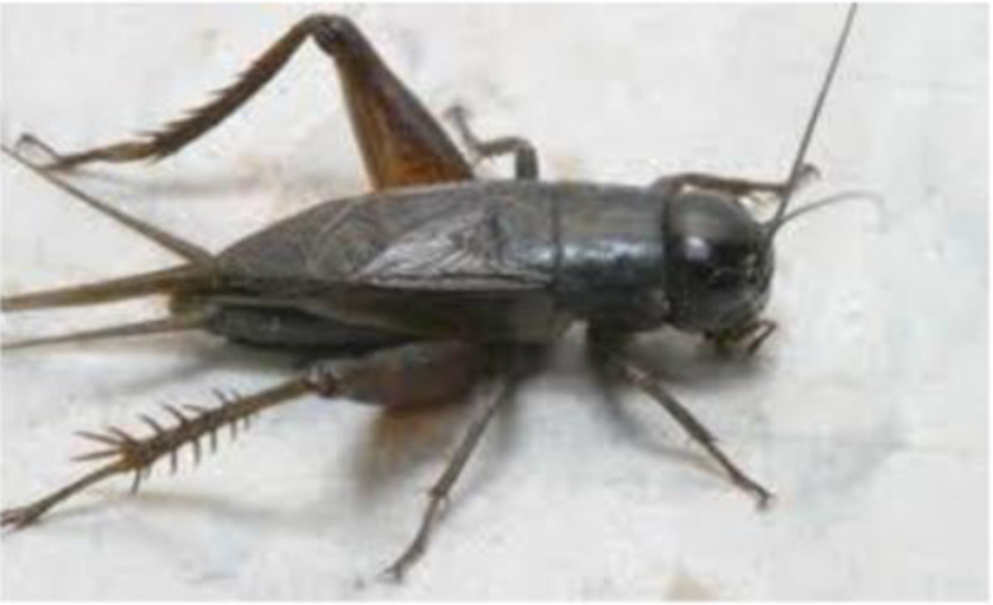
*Gryllus similis* male. Source: Anankware et al. [(101), p. 36].

**Figure 4:**
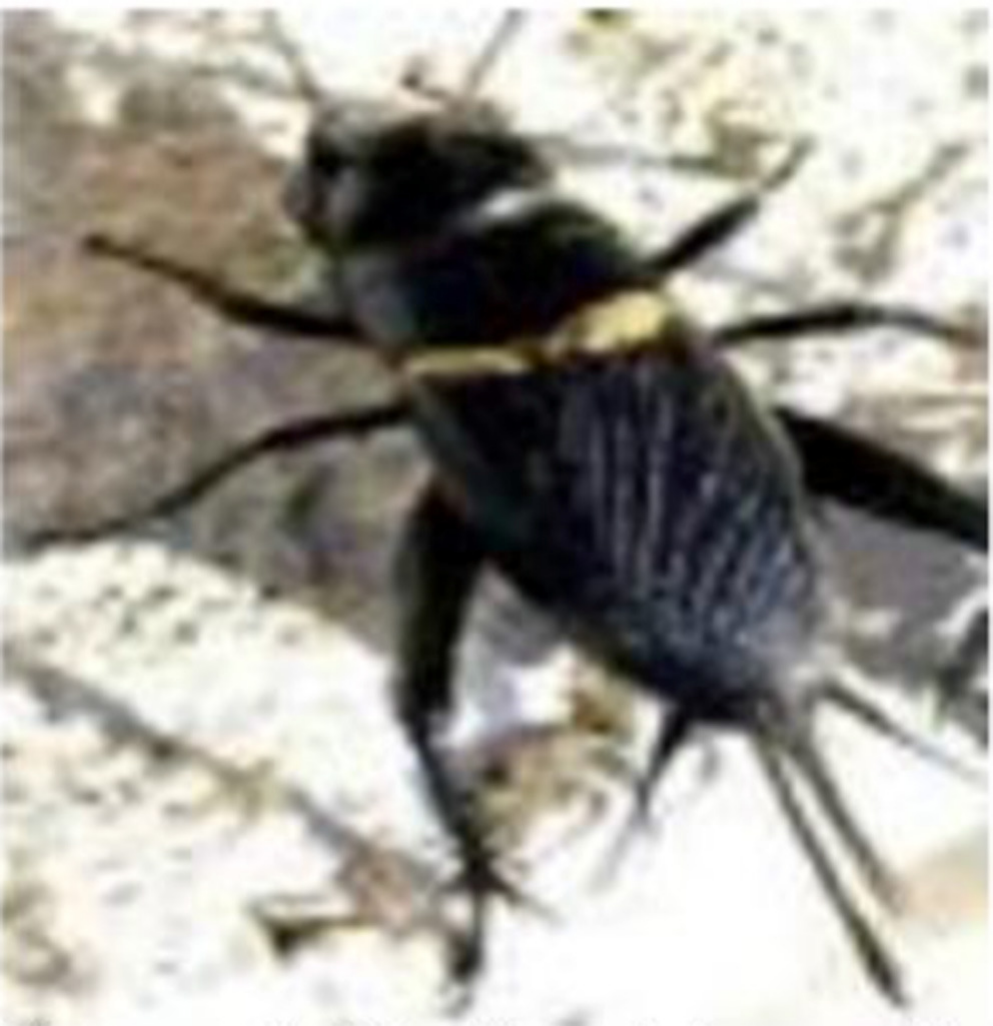
*Gryllus bimaculatus*. Source: Orinda [(29), p. 15].

**Figure 5:**
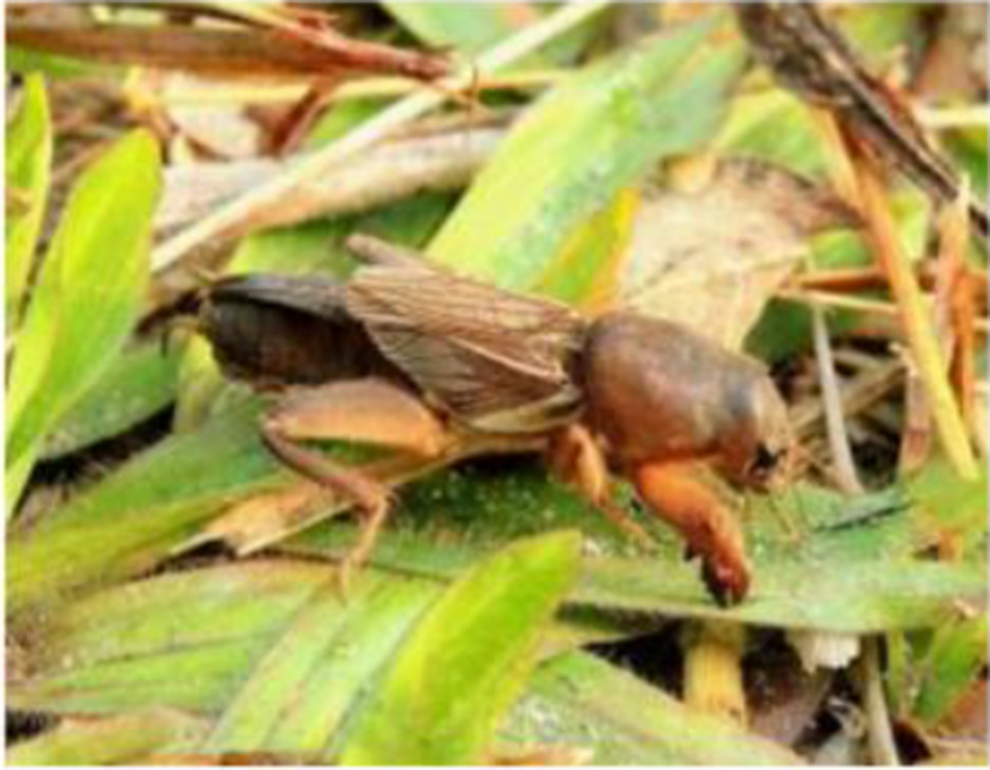
*Gryllotalpa orientalis*. 15, February, 2014. Photograph by Michael Kesil.

**Figure 6:**
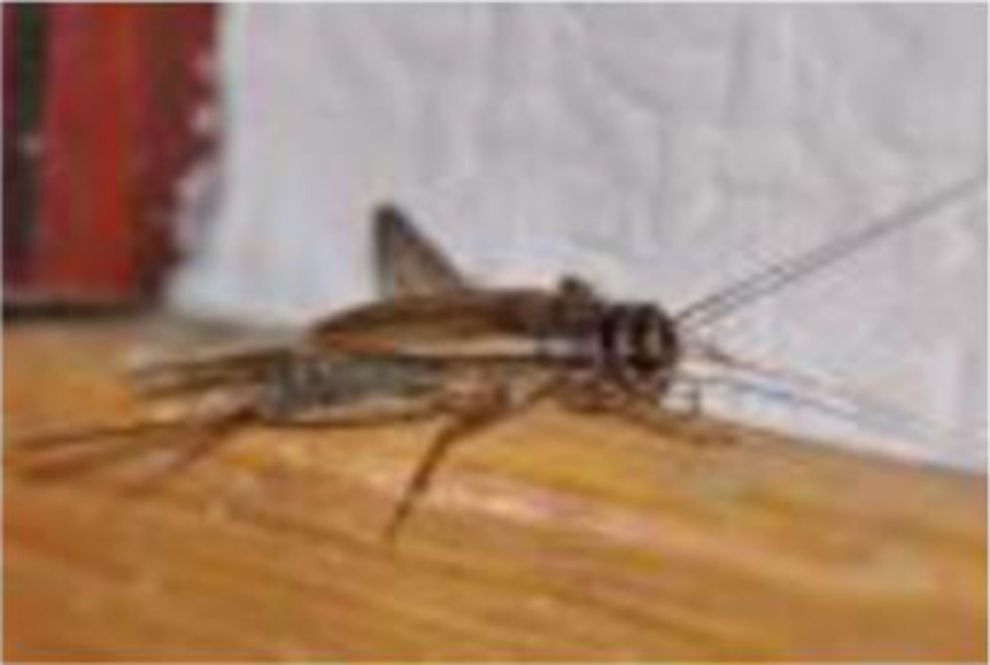
*Acheta domesticus*. 29 August, 2010. Courtesy of Aiwok.

**Figure 7:**
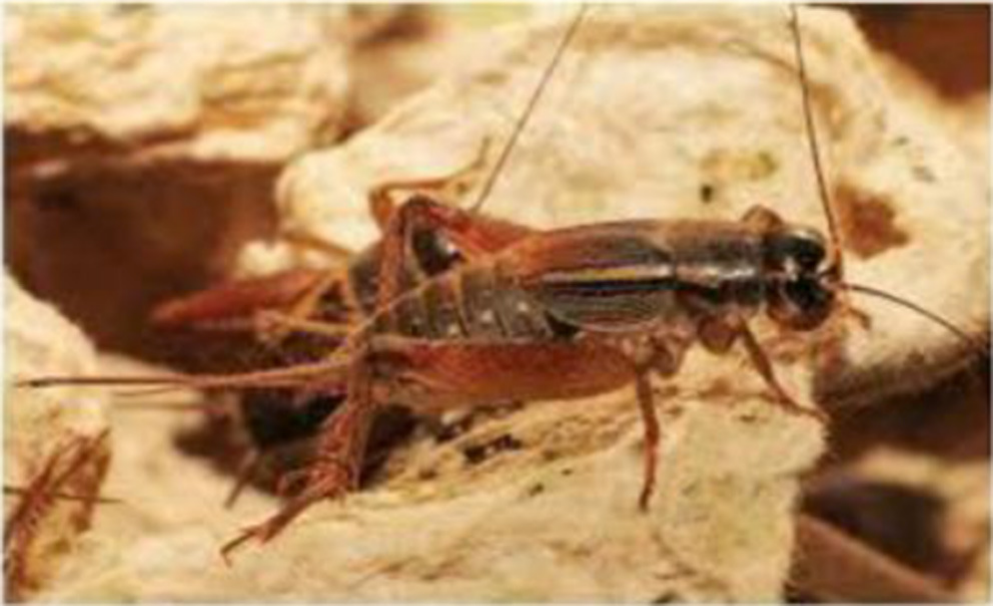
*Scapsipedus icipe*. Source: Magara et al. [(28), p.2].

The rapid day-to-day global population increase is predicted to reach 9.74 billion people by the year 2050 (1). This population growth requires an urgent intervention to increase food production to keep it in tandem with the expanding demand (2). As it is, food production may not meet demand because of the increasing scarcity of the necessary arable land. This situation is exacerbated by climate change, lack of water, and poverty (3). This therefore calls for a shift toward alternative and novel food production systems that are cheap, environmentally friendly, adaptable to climate change, and sustainable. One of the promising options is entomophagy, which is defined as the practice of eating insects (4–8). Entomophagy is a common practice in many parts of the world, and there are 2,100 species of insects that are consumed as food in over 110 countries (9). Out of this number, 500 insect species are consumed in Africa (10–13), 324 insect species are consumed in China (14–22), 255 insect species are consumed in India (23, 24), and over 164 species of insects are consumed in Thailand (25, 26). The commonly consumed insects include the orders Coleoptera, Lepidoptera, Hymenoptera, Orthoptera, and Hemiptera, respectively (27). Among the Orthopterans, crickets stand as the most-consumed insects across the globe (28–30) (Figure 1). Both the nymph and adult stages of crickets are consumed as food (27, 31). The most common species usually reported include *Brachytrupes membranaceus* (Figure 2), *Gryllus similis* (Figure 3), *Gryllus bimaculatus* (Figure 4), *Gryllotalpa orientalis* (Figure 5) and *Acheta domesticus* (Figure 6) (29, 32–36). However, this may not be representative of the exhaustive number of crickets that are edible globally. Moreover, a more recent discovery of a new edible cricket *Scapsipedus icipe* (Figure 7) (37, 38) in Africa makes us conclude there may be other edible crickets that have not yet been documented, and this forms a basis for our review (Figure 2).

Jongema (9) documented edible insects around the globe. The database contains several species and covers several orders of edible insects, including edible crickets. Such a database provides valuable information that could further be improved upon with additional data on the nutritional content of these edible insects, their sensory attributes, and the potential risks to potential consumers. Information on whether these insects can be farmed and the overall benefits to consumers are crucial.

Crickets have been consumed as food in Asia, Latin America, and Africa as far back as prehistoric times. In Biblical scriptures, cricket consumption is recommended to the Israelites by God to be fit for their consumption: “these you may eat any kind of locust, katydid, cricket or grasshopper” (Leviticus 11: 22). In China, crickets have been consumed as food for over 2,000 years (14, 39). In Africa, crickets have formed a daunted cuisine and a valuable complement of food enrichment for many years (32, 40–43). In recent years, consumption of edible crickets has become more appreciated in Europe, America, and Australia with the recognition of its nutritional benefits and food security (44–47).

The high nutritional content with the presence of protein, essential amino acids, lipids, the monounsaturated fatty acids (MUFA) and polyunsaturated fatty acids (PUFA), mineral elements, carbohydrates, energy, and the ease of farming make crickets promising as a sustainable food source (29, 48). The rearing of crickets as mini livestock seems to be more ecofriendly because of their low emission of greenhouse gases, low water and feed intake, and the small land requirement for their production as compared to livestock (29, 30, 49). Crickets also show higher feed conversion efficiency when compared to mammalian livestock. For instance, van Huis et al. (50) reported the feed conversion efficiency of *A. domesticus* to be two-fold as compared to that of broiler chickens and pigs, four-fold compared to that of sheep, and more than 12-fold compared to that of cattle. Moreover, crickets may be produced on locally available food substrates such as agro byproducts and weeds, and they thus aid in cleaning the environment (28, 29, 51). In recent years, research on using crickets as human food and feed has increased with the recognition of cricket nutritional benefits and their potential of ensuring food security (27, 44, 45). Globally, the most frequently consumed cricket family is Gryllidae followed by the Gryllotalpa family (9, 52). House cricket *Acheta domesticus* Linnaeus forms the most-consumed cricket species worldwide.

While edible crickets are found to be rich sources of proteins and other nutrients (5, 53), there remain challenges and scientific knowledge gaps that need to be filled. One of the challenges for promoting edible crickets for human food is the lack of knowledge of the particular species that are edible and where they are found in the globe. The overall goal of this review is consequently to offer exact information concerning the number of crickets that are edible in the world, their nutrition content, sensory attributes, the possibility of being farmed, the safety of consumers and other benefits one can draw from them.

## The Global Distribution of Edible Crickets

Crickets are non-wood wild products found in natural resources all around the globe apart from cooler regions at latitudes 55° and beyond; the greatest species abundance is found in the tropics where temperatures are warm and suitable for their faster development compared to cold regions (54). Crickets occur in the various habitats that include grassland, bushes, forests, trees, marshes, beaches, caves, underground, and in buildings (55). The edible crickets in this review belong to the infraorder Gryllidea that comprise the families Gryllotalpidae, Trigonidiidae, Gryllidae, and Phalangopsidae. Although more than 6,000 Gryllidea have been described (56), the actual number of crickets that are edible in this group is not known. In this review, we report 62 cricket species that are consumed as human food or used as livestock feed in different parts of the world (Supplementary Table 1). The consumption of the crickets depends on their distribution and the cultural appropriateness of cricket consumption to people in a particular country. The distribution of these edible crickets in five continents is as follows: Asia (41 species), Africa (26 species), America (five species), Europe (four species), and Australia (four species) (Supplementary Table 1). Africa tops the list with 25 countries that consume various crickets, followed by Asia (13 countries), America (five countries), Europe (four countries), and Australia (two countries) (Supplementary Table 1). Our review shows that crickets are more consumed in developing countries that are experiencing food insecurity than in developed countries. However, the consumption of crickets as food and feed is starting to take off in western countries despite the early stigma that has depicted insect consumption as a poor man's food in developing countries. This trend is changing rapidly as legislations have been put in place in some western countries, recognizing edible crickets as novel resources to mitigate food insecurity and malnutrition (44).

## The Nutritional Composition of Different Species of Edible Crickets

Edible crickets are excellent sources of proteins, lipids, carbohydrates, mineral salts, and vitamins (Table 1). However, the nutritional composition of these crickets varies across the different species (29, 66, 78). The nutritional content can also vary within the same species of cricket influenced by the stage of development, habitat, climate, sex, and the food substrate fed on by the cricket (71, 79). The nutritional value may also be influenced by the method in which the crickets are processed (i.e., drying, cooking, smoking, deep-frying, roasting, and toasting) before consumption (50, 80). Most of the edible crickets supply adequate energy and proteins to the consumer diet, at the same time meeting the amino acid requirements. Crickets also possess a high value of monounsaturated (MUFA) and polyunsaturated fatty acids (PUFA) (59, 67, 68). Besides, these insects are rich in micro-nutrient elements such as calcium, potassium, magnesium, phosphorus, Sodium, Iron, zinc, manganese, and copper as well as vitamins like folic acid, pantothenic acid, riboflavin, and biotin, which are the most deficient nutrients in humans (5, 29, 66). This therefore implies that crickets are a good source of various nutrients required by humans for proper growth and development. The following subsections provide details of the specific nutritional composition of edible crickets.

**Table 1:** The nutritional composition of different species of edible crickets and selected animal tissues.

**Cricket species**	**Stage**	**Protein (g/100 g dry weight)**	**Lipid (g/100 g dry weight)**	**Fiber (g/100 g dry weight)**	**Ash (g/100 g dry weight)**	**Carbohydrates (g/100 g dry weight)**	**Energy value (kcal/100 g dry matter)**	**References**
*Acheta domesticus*	Nymph and Adult	62.41–71.09 NR	9.80–22.8 19.20–29.58	10.20 NR	5.10–9.10 NR	NR NR	455.19 NR	(5, 29, 57, 58)
*Gryllus assimilis*	Adult Nymph	56.00 ± 3.10 55.60 ± 1.10 65.52 ± 1.39 71.04 ± 0.01 56.4	21.80 ± 2.65 11.90 ± 0.50 7.00 ± 0.12 NR 34.00	8.28 8.00 7.00 NR NR	6.40 NR NR NR 4.08 ± 0.43	12.46 ± 0.16 8.60 ± 1.49 NR NR NR	397.00 ± 1.69 NR NR NR 537.50	(59–65)
*Gryllus bimaculatus*	Adult	57.49–70.10	14.93–33.44	9.53 ± 0.46	NR	NR	120.00	(29, 66)
*Brachytrupes spp*	Adult	65.35 ± 0.36	11.76 ± 0.63	13.29 ± 1.61	4.88 ± 0.23	2.50 ± 0.85	536.42 ± 0.47	(67)
*Gryllus testaceus*	Adult	58.30 ± 0.91	10.30 ± 0.31	10.40	2.96 ± 0.09	NR	18.10	(51, 68, 69)
*Tarbinskiellus portentosus*	Adult	58.00 ± 0.05	23.70 ± 0.05	1.16 ± 1.01	7.93 ± 0.04	NR	460.82	(70)
*Gryllodes sigillatus*	Nymph	56.00	NR	NR	NR	NR		(60, 61)
*Teleogryllus emma*	Adult	55.65 ± 0.28	25.14 ± 0.11	10.37 ± 0.19	10.37 ± 0.19	NR		(66)
*Brachytrupes membranaceus*	Adult Nymph	53.4 ± 0.19	15.80 ± 0.23	6.30 ± 0.14 5.0 ± 0.30	6.00 ± 0.12 3.23 ± 0.01	15.10 ± 0.22	454.7 ± 2.25	(17, 71, 72)
*Brachytrupes portentosus*	Adult	48.69 ± 0.25 NR	20.60 ± 0.60 NR	11.61 ± 0.20 0.5–8.3	5.40–20.50 9.36 ± 0.34	NR NR	90.06–134.0 NR	(73, 74)
*Gryllotalpa africana*	Adult	22.0 ± 0.86	10.80 ± 1.24	7.4 ± 0.24	12.60 ± 0.97	47.20 ± 0.32	362.3 ± 2.34	(71)
*Acheta testacea*	Adult	18.6	6.00	NR	NR	NR	133.00	(75, 76)
*Acheta confirmata*	Adult	NR	21.14	NR	NR	NR	NR	(61)
**Animal tissue**								
Goat, roasted		27	3	0	NR	0	143	(77)
Broiler		24	14	0	NR	0	165	(77)
Pork		27	6.00	0	NR	1.5	242	(77)

### Protein Composition of Different Species of Edible Crickets

Previous studies have reported protein valuea for 14 edible cricket species, ranging from 18.6 to 71.1% in dry weight (29, 57, 66, 81) (Table 1). The protein content of the different cricket species is within the range of the reported protein for other edible insects, including other Orthopterans (5). The variation in protein content observed in the crickets could be due to the influence of the species, diet, habitat, and the stage of development of the examined cricket. Compared to the protein content of the common meat sources listed in Table 1, most of the edible crickets have a higher protein content than that of the roasted goat, broiler chicken, and pork. The protein digestibility of some crickets was also investigated in a review and was 50.2% for *Brachytrupes* sp. and 83.9% for *A. domesticus* (82, 83). These protein digestibility values for the crickets are slightly lower compared to values for eggs (95%), beef (98%), and cow milk (95%) (84). On the other hand, the protein digestibility values for the crickets are higher than those of many plant proteins, such as sorghum (46%), maize (73%), wheat (81%), and rice (66%) (85). The amounts of nitrogenous substances in crickets may, however, be higher than their actual protein content since some nitrogen is also bound in the exoskeleton (29, 86).

### Lipids Composition of Different Species of Edible Crickets

Edible crickets contain, on average, 4.30–33.44% of lipids in dry matter basis (Table 1). In some cricket species, the lipids content are higher in the nymphal stages than in adults (65, 87), while in other species they are lower in nymphs compared to the adult stage (29, 58). *Gryllus bimaculatus and A. domesticus* are among those cricket species with the highest lipid content.

Edible crickets have two different forms of lipids, namely, triglycerols and phospholipids. Triglycerols are ~80% of lipids. They store energy that is required for activities that require high energy intensity in the cricket, such as longer flight and hopping. This energy can be available for humans after feeding on crickets. Phospholipids are the second most dominant group of lipids. Their value in cricket lipids is usually <20%, but their variation is dependent on the stage of development of the cricket and cricket species (88, 89). The role of phospholipids in the structure of cell membranes has been studied (88). Crickets are richer in lipid content when compared to goat, chicken, and pork meats (Table 1).

### Ash Composition of Different Species of Edible Crickets

Edible crickets possess a significant amount of ash, ranging from 2.96 to 20.50 mg/100 g dry weight (Table 1). The lowest ash content was reported by Wang et al. (68) for *G. testaceus*. The low ash content of *G. testaceus* implies low mineral content. On the other hand, the highest ash content was reported by McDonald et al. (74) for *B. portentosus*. The higher the ash content the higher the value of the mineral elements for human health. Crickets have a higher content of ash when compared to goat, broiler, and pork meat (Table 1).

### Fiber Composition of Different Species of Edible Crickets

Crickets contain a significant quantity of fiber that ranges between 0.5 and 13.4% (Table 1). The insoluble chitin in the exoskeleton of the edible crickets forms a major part of fiber (50, 90). In commercially farmed crickets, the chitin ranges from 2.7 to 49.8 mg per kg of fresh weight and 11.6 to 137.2 mg per kg of dry weight (91). People from tropical countries can digest chitin by the help of a bioactive chitinase enzyme, which has developed in their gastric juices as a result of consuming edible crickets in their regular diet unlike people from outside the tropics (92). To enable people from outside the tropics to consume crickets without any complication, the chitin must be removed (91).

The chitin plays a significant role in protecting crickets against some parasitic attacks and allergic states (91, 93). Lee et al. (94) also reported that chitin is antivirally active against tumor formation. Chitin and its associated derivative chitosan have a functional role that could improve retinol and 6.8–8.2 μg of β-carotene per 100 g of dry weight. The chitin of the crickets has also been shown to influence the gut microflora, which plays an important role in the health of human beings and other animals, such as dogs (95, 96). Crickets are richer in fiber compared to the other meat sources (Table 1).

### Carbohydrate Composition of Different Species of Edible Crickets

Carbohydrate is a major source of energy, though crickets do not require it for their growth since they can synthesize it from amino acids and lipids in their bodies (97). The carbohydrate content of edible crickets reported in the previous studies ranged from 2.50–47.20 g/100 g of dry weight (Table 1). The carbohydrate content in crickets is greatly influenced by the diet they consume (98). Crickets store their carbohydrates in the fat body, mainly in the form of glycogen, which can be later rapidly hydrolyzed into a readily useable form of energy: trehalose. The utilization of carbohydrates as a source of energy in insects is mostly relevant during metamorphosis due to metabolic interconversions (98) as well as during male stridulation in crickets (99, 100). By feeding on the crickets or their byproducts, we can obtain and make use of these carbohydrates. The highest amount of carbohydrates is reported in the mole cricket *Gryllotalpa africana* while the lowest carbohydrate content is found in *Brachtrupes* sp. Edible crickets are a good source of carbohydrates when compared to goat, broiler, or pork meat (Table 1).

### Energy Content of Different Species of Edibles Crickets

Different studies have reported the caloric energy content of 12 cricket species, which ranges from 18 to 536 kilocalories (kcal) per 100 g of dry weight (Table 1). The energy value of the crickets is influenced by the species, lipid content, and their stage of development. *Gryllus assimilis* and *Brachytrupes* sp. have the highest energy content while *Gryllus testaceus* has the lowest. Furthermore, the review shows that the *Gryllus assimilis* nymphal stage has more energy than the adult stage (60, 61) (Table 1). On one hand, the calorific energy content for six cricket species (61, 62, 67, 70) is within the range of 293 and 762 kcal per 100 g of the dry weight of the other edible insect species (83). On the other hand, the calorific energy content of the five other crickets (Table 1) was low (65, 68) compared to the description obtained by Ramos-Elorduy et al. (83) for the other edible insect species. This data varied—most probably because of the variation of the cricket species and the method of analyzing their nutritional content. When the crickets are compared with the goat, broiler, or pork meat, most of the crickets have a higher energy content than these other meat sources (Table 1). This finding shows that the crickets are an energy-rich food source for humans.

### Amino Acids Composition of Different Species of Edible Crickets

Edible crickets are rich in amino acids, which vary across the cricket species (Table 2). Glutamic acid is the most abundant amino acid in *T. portentosus, G. assimilis, G. testaceus, A. testacea, G. bimaculatus*, and *A. domesticus*, while leucine is the most abundant amino acid in *G. sigillatus*. The most abundant essential amino acids in these crickets are valine, ranging from 1.07 to 11.45 g/100 g of dry matter, leucine, ranging from 3.97 to 9.75 g/100 g of dry matter, and lysine, ranging from 2.42 to 7.90 g of dry matter (Table 2). The extensive variation of the amino acids amongst the edible crickets could be due to the variation in the diet they consume, stage of development, species, sex, habitat, and measuring methods (110, 111). Compared to amino acids from livestock meats in Table 2, crickets such as *T. portentosus, G. sigillatus*, and *G. assimilis* have more valine amino acid than pork and broiler chicken and similar content of all other amino acids (108). On the other hand, *T. portentosus, G. sigillatus, G*. testaceus, and *A. domesticus* have a higher content of phenylalanine than chicken (112) but similar content to that of pork (106).

**Table 2:** Amino acid composition (g/100 g protein) of different species of edible crickets and selected animal tissue (g/100g protein) and amino acid score.

**Amino acid**	**Cricket species**							**Animal tissue**		**Amino acid Score**	
	***Tarbinskiellus portentosus***	***Gryllodes sigillatus* nymph**	***Gryllus assimilis***	***Gryllus testaceus***	***Acheta testacea***	***Gryllus bimaculatus***	***Acheta domesticus***	**Pork loin muscle**	**Broiler**	**Infants**	**Children and adult**
**Essential Amino acids**											
Valine	11.45 ± 0.98	5.20	4.62 ± 0.59	4.42 ± 0.00	3.44	3.20 ± 0.03	1.07	3.36–3.62	3.36–4.58	1.0–1.1	1.3–1.4
Isoleucine	3.03 ± 0.19	3.70	2.12 ± 0.73	3.09 ± 0.00	2.98	2.16 ± 0.04	4.45 ± 0.21	3.69–4.80	3.09–4.23	0.85–0.89	1.4–1.5
Leucine	ND	6.90	7.74 ± 0.64	5.521 ± 0.13	6.09	3.97 ± 0.05	9.75 ± 0.35	6.50–7.36	5.12–6.88	0.84–0.92	1.2–1.3
Lysine	6.10 ± 0.07	5.30	7.90 ± 0.64	4.79 ± 0.10	4.61	2.42 ± 0.01	5.40 ± 0.00	7.80–8.78	5.81–7.77	0.78–1.0	1.0–1.2
Threonine	3.81 ± 0.21	3.50	3.55 ± 0.63	2.75 ± 0.12	2.90	2.00 ± 0.04	3.60 ± 0.00	3.37–5.11	2.78–3.66	0.90–0.98	1.3–1.4
Phenylalanine	2.59 ± 0.13	3.10	0.72 ± 0.20	2.86 ± 0.06	NR	1.83 ± 0.01	3.00 ± 0.28	2.83–3.98	2.33–2.49	NR	NR
Methionine	2.42 ± 0.09	1.60	0.63 ± 0.20	1.93 ± 0.06	NR	0.27 ± 0.01	1.40 ± 0.14	2.36–2.86	1.40–2.08	NR	NR
Histidine	ND	2.20	1.32 ± 0.37	1.94 ± 0.01	1.54	2.50 ± 0.08	2.25 ± 0.07	3.46–3.63	2.47–4.44	1.1–1.2	1.2–1.3
Trypatophan	1.35 ± 0.23	0.90	0.95 ± 0.20	NR	2.44	NR	0.55 ± 0.07	0.82–1.33	1.05–1.11	0.40–0.57	0.8–1.1
Methionine and Cystine	NR	NR	NR	NR	3.09	NR	NR	3.35–4.00	2.22–3.36	0.70–0.84	0.86–1.0
Phenylalanine and Tyrosine	NR	NR	NR	NR	6.24	NR	NR	4.63–5.89	4.28–6.01	0.90–1.1	1.6–2.0
**Non-essential amino acids**											
Tyrosine	4.73 ± 0.13	4.20	5.44 ± 0.66	3.94 ± 0.02	NR	2.73 ± 0.02	1.00	1.80–1.91	1.95–3.52	NR	NR
Arginine	0.32 ± 0.31	5.70	3.02 ± 1.36	3.68 ± 0.12	4.51	3.60 ± 0.04	6.10 ± 0.00	4.60–6.62	3.76–7.08	NR	NR
Aspartic acid	6.99 ± 0.97	7NR	8.64 ± 0.63	3.72 ± 0.07	6.92	3.60 ± 0.04	7.75 ± 0.92	7.26–8.09	5.96–7.89	NR	NR
Glutamic acid	19.24 ± 1.32	NR	2.41 ± 0.14	9.07 ± 0.31	9.68	6.39 ± 0.07	10.45 ± 0.07	12.9–13.3	9.35–11.03	NR	NR
Serine	3.17 ± 0.69	NR	0.61 ± 0.20	3.72 ± 0.07	3.59	2.73 ± 0.01	1.02	3.11–3.30	2.58–3.06	NR	NR
Aspargine	3.27 ± 0.52	NR	NR	6.290 ± 0.2	NR	NR	NR	NR	NR	NR	NR
Glycine	NR	NR	0.36 ± 0.73	3.62 ± 0.11	4.72	3.32 ± 0.01	1.04	2.99–3,14	3.44–3.75	NR	NR
Alanine	0.14 ± 0.02	NR	4.02 ± 0.63	5.55 ± 0.09	7.80	5.64 ± 0.01	8.85 ± 0.07	3.93–4.21	3.79–4.91	NR	NR
Cysteine	NR	0.90	0.74 ± 0.14	1.01 ± 0.02	NR	5.10 ± 0.00	0.8 ± 0.00	0.99–1.14	0.82–1.28	NR	NR
Proline	1.44 ± 0.39	NR	1.26 ± 0.73	4.50 ± 0.08	4.52	1.99 ± 0.01	1.15	2.99–3.14	1.94–1.98	NR	NR
Taurine	1.25 ± 0.43	NR	NR	NR	NR	NR	141.00	NR	NR	NR	NR
Ornithine	3.10 ± 2.96	NR	NR	NR	NR	NR	NR	NR	NR	NR	NR
EAA	35.48	NR	NR	32.25	NR	21.08	NR	NR	NR	NR	NR
NEAA	66.16	NR	NR	36.42	NR	32.75	NR	NR	NR	NR	NR
EAA/NEAA	0.54	NR	NR	0.89	NR	0.64	NR	NR	NR	NR	NR
Total amino acids	101.64	43.20	56.49	68.67	NR	53.83	NR	NR	NR	NR	NR
References	(70)	(60, 61)	(102)	(68, 103)	(76)	(66)	(104, 105)	(106, 107)	(107, 108)	(107, 109)	(107, 109)

*NR, not reported; nd, not detected; EAA, Essential Amino Acids; NEAA, Non Essential Amino Acids*.

Some crickets possess high values of lysine, tryptophan, and threonine, which are lacking in some of the cereal proteins that are major parts of the daily diets of many households. For instance, in Africa, where malnutrition is rampant, the consumption of crickets such as *A. domesticus, G. bimaculatus*, and *G. assimilis* can help mitigate deficiencies in the required amino acids (66, 102, 104). In Australia, the people of Papua New Guinea consume tubers as food, which contain low values of the lysine and leucine. The resulting nutritional deficiency can, therefore, be solved through consuming nymphs and adults of the mole cricket *Gryllotalpa* species and *A. domesticus* and *G. bimaculatus* as food (66, 104, 113) with high quantities of lysine. On the other hand, tubers contain a high proportion of tryptophan and aromatic amino acids, which are available in small quantities in the nymphs and adult crickets. It is therefore advisable to consume a mixed diet of tubers and crickets to have a balance in the required amino acid (50, 114). When the protein of about 100 edible insect species was analyzed, the content of essential amino acids was found to be ranging between 46 and 96 g/100 g dry matter of the total amount of amino acids (87). This implies that the crickets in this review are rich sources of amino acid for humans.

### Fatty Acids Composition of Different Species of Edible Crickets

The edible crickets in this review possess higher contents of oleic, linoleic, linolenic, stearic (C18 fatty acids), and palmitic acid (C16 fatty acid) as compared to other fatty acids (66, 68, 73, 115), (Table 3). Linoleic acid, ranging from 4.15 to 41.39 g/100 g of dry matter, is the most abundant fatty acid in *T. portentosus, G. testaceus, G. assimilis, A. domesticus, G. bimaculatus, T. emma*, and *A. confirmata*. On the other hand, oleic (38.27 g/100 g of dry matter) and arachidonic acid (50.43 g/100 g of dry matter) are the most abundant fatty acids in *Brachytrupes* sp. and *B. portentosus*, respectively (Table 3). The second and third most abundant fatty acid in various crickets is as follows: in *T. portentosus*, we have pentadecanoic and myristic acid; in *G. testaceus* we have oleic and palmitic acid, in *G. assimilis* we have palmitic and oleic acid, in *A. domesticus* we have palmitic and oleic acid, in *G. bimaculatus* we have oleic and palmitic acid, in T. emma we have oleic and palmitic acid, in *A. confirmata* we have oleic and myristic acid, in *Brachytrupes* sp. we have linoleic and palmitic acid, and in *B. portentosus* we have stearic and Eicosatrienoic acid. The variation in fatty acid values in the crickets can be attributed to the difference in species, stage of development, diet, and environmental conditions in different localities (29, 58, 114).

**Table 3:** Fatty acid composition (g/100 g DM) of different species of edible crickets.

**Fatty acid**	**Cricket species**										**Animal tissue**	
	***Tarbinskiellus portentosus***	***Gryllus testaceus***	***Gryllus assimilis***	***Acheta domesticus***	***Gryllus bimaculatus***	***Teleogryllus emma***	***Brachytrupes* sp.**	***Brachytrupes portentosus***	***Acheta testacea***	***Acheta confirmata***	**Pork loin**	**Broiler**
Lauric acid (C12:0)	1.16 ± 0.16	0.54 ± 0.04	0.12 ± 0.00	0.10 ± 0.00	0.04	0.02	NR	NR	NR	NR	0.21	NR
Tridecanoic acid (C13:0)	NR	NR	0.02 ± 0.01	NR	0	0	NR	NR	NR	NR	NR	NR
Myristic acid (C14:0)	6.74 ± 0.47	0.39 ± 0.02	1.28 ± 0.01	0.44 ± 0.00	0.05	0.18	0.96 ± 0.01	Nd	NR	26.10	1.3–1.4	0.45–0.69
Pentadecanoic acid (C15:0)	16.74 ± 1.33	NR	0.37 ± 0.01	0.11 ± 0.00	0.01	0.02	NR	Nd	NR	NR	4.1–4.7	NR
Palmitic acid (C16:0)	NR	10.18 ± 0.20	25.85 ± 0.06	22.65 ± 0.37	2.16	3.06	21.31 ± 0.49	1.61 ± 0.05	NR	5.50	23.2–27.3	23.8–24.9
Heptadecanoic acid (C17:0)	NR	NR	0.57 ± 0.01	0.12 ± 0.00	0.03	0.04	NR	0.13 ± 0.02	NR	NR	0.2–0.3	NR
Stearic acid (C18:0)	NR	2.63 ± 0.09	14.07 ± 0.03	8.54 ± 0.00	0.76	0.07	12.24 ± 0.24	35.79 ± 0.02	NR	1.20	12.2–16.1	5.7–5.9
Arachidic acid (C20:0)	NR	NR	0.56 ± 0.01		0.12	0.09	0.49 ± 0.01	Nd	NR	NR	NR	NR
Heneicosanoic acid (C21:0)	NR	NR	0.03 ± 0.00	0.24 ± 0.00	0.04	0.04	NR	NR	NR	NR	NR	NR
Behenic acid (C22:0)	2.34 ± 0.27	NR	0.57 ± 0.00		0.03	0.01	NR	NR	NR	NR	NR	NR
Tricosanoic acid (C23:0)	NR	NR	0.22 ± 0.01	0.02 ± 0.04	0	0.07	NR	NR	NR	NR	NR	NR
Lignoceric acid (C24:0)	NR	NR			0.01	0.01	NR	NR	NR	NR	NR	NR
Myristoleic acid (C14:1)	NR	NR	0.06 ± 0.01	0.44 ± 0.00	0	0.02	NR	NR	NR	NR	NR	NR
Palmitoleic acid (C16:1)	NR	3.11 ± 0.10	1.92 ± 0.01	0.34 ± 0.00	0.17	0.91	0.96 ± 0.00	0.71 ± 0.03	NR	2.40	2.1–2.8	7.1–7.4
Heptadecenoic acid (C17:1)	NR	NR	0.19 ± 0.00	0.24 ± 0.00	0.01	0.03	NR	NR	NR	NR	NR	NR
cis Oleic acid (C18:1n-9)	NR	29.58 ± 0.20	25.03 ± 0.11	20.18 ± 0.02	2.91	6.98	38.27 ± 0.67	3.4 ± 0.03	NR	31.10	32.8–43.7	40.3–40.9
Eicosenoic acid (C20:1)	NR	NR	0.24 ± 0.00	NR	0.03	0.04	NR	NR	NR	NR	NR	NR
Erucic acid (C22:1n-9)	NR	NR	0.05 ± 0.01	0.52 ± 0.01	0.01	0.04	NR	NR	NR	NR	NR	NR
cis Linoleic acid (C18:2n-6)	18.94 ± 0.02	37.82 ± 0.20	26.13 ± 0.18	41.39 ± 0.29	4.15	9.61	22.14 ± 0.59	NR	NR	32.20	10.7–14.2	16.2–17.5
Eicosadienoic acid (C20:2)	NR	NR	1.60 ± 0.01	0.00	0.04	0.02	NR	NR	NR	NR	NR	NR
Eicosatrienoic (C20:3n-3)	NR	NR	0.01 ± 0.00	NR	NR	NR	NR	NR	NR	NR	NR	NR
Eicosatetraenoic (C20:4n-3)	NR	NR	0.21 ± 0.02	NR	NR	NR	NR	NR	NR	NR	NR	NR
Docosadienoic acid (C22:2n-6)	NR	NR	0.03 ± 0.02	0.11 ± 0.01	0.02	0.01	NR	NR	NR	NR	NR	NR
Linolenic acid (C18:3n-6)	NR	10.12 ± 0.10	NR	1.11 ± 0.00	0.01	0	2.55 ± 0.18	NR	NR	NR	NR	NR
Alpha-linolenic acid (C18:3n-3)	NR	NR	NR	NR	0.08	0.22	NR	Nd	NR	1.70	1.0–1.1	0.77–0.85
Eicosatrienoic acid (C20:3n-6)	NR	NR	NR	0.01 ± 0.02	0.02	0.01	NR	7.94 ± 0.04	NR	NR	NR	NR
Arachidonic acid (C20:4n-6)	0.55 ± 0.28	NR	NR	0.01 ± 0.02	0.01	0.27	NR	50.43 ± 0.55	NR	NR	0.1–0.2	0.76-0.97
Eicosapentaenoic (C20:5n-3)	NR	NR	NR	0.01 ± 0.02	0	0.01	NR	Nd	NR	NR	0.2-0.4	0.05–0.07
SFA	50.58	13.74	43.72	32.22	3.25	3.61	34.99 ± 0.24	37.54 ± 0.08	36.5	32.80	40.7	30.9–32.2
MUFA	28.98	32.69	27.49	21.72	3.13	8.02	39.23 ± 0.66	4.11 ± 0.06	30.1	33.50	47.2	48.0–49.1
PUFA	20.32	47.94	28.80	42.64	4.33	10.15	24.68 ± 0.77	58.37 ± 0.59	31.1	33.90	11.7	19.1–20.4
TUFA	49.30	80.63	56.29	64.36	7.46	18.17	63.91	62.48	61.20	67.40	58.90	67.10–69.50
PUFA/SFA ratio	0.40	3.49	0.66	1.32	1.33	2.81	0.71	1.55	0.86	1.03	0.6	0.61–0.66
n-3	NR	NR	1.99	0.01	0.08	0.23	NR	NR	NR	NR	1.2–1.5	0.82–0.93
n-6	19.49	47.94	26.81	42.63	4.25	9.92	24.69	58.37	NR	NR	10.8–14.4	17.8–19.1
EFA	18.94 ± 0.02	37.82 ± 0.20	26.13 ± 0.18	41.39 ± 0.29	4.23	9.83	22.14 ± 0.59	NR	NR	33.90	11.70–15.30	16.20–18.35
References	(70)	(68)	(59)	(115)	(66)	(66)	(67)	(73)	(76)	(61)	(106)	(112)

*NR, not reported; nd, not detected; SFA, saturated fatty acid; MUFA, monounsaturated fatty acid; PUFA, polyunsaturated fatty acid; UFA, unsaturated fatty acids; EFA, essential fatty acids*.

This review demonstrates that the different cricket lipids are highly unsaturated, with either linoleic and oleic or linoleic and pentadecanoic acid or arachidonic and eicosatrienoic acid being the most abundant unsaturated fatty acids and palmitic, myristic, and stearic acids being the most abundant saturated fatty acids. Linoleic, oleic, myristic, Pentadecanoic, stearic, and palmitic acid are also predominant in other edible insects, including other Orthopterans (5, 107). These fatty acids are also the most abundant in livestock meat, including chicken and pork (106, 107, 112). *Tarbinskiellus portentosus, G. testaceus, G. assimilis, A. domesticus, A. confirmata, Brachytrupes* sp., and *B. portentosus* have higher content of polyunsaturated fatty acids (PUFA) compared to pork and broiler chicken meat (Table 3). *Gryllus bimaculatus* and *T. emma*, on the other hand, have lower content of polyunsaturated fatty acids (PUFA) compared to pork and broiler chicken meat. Most of the crickets in our review, except for *G. bimaculatus* and *T. emma*, have more essential fatty acids than the pork and broiler chicken. Crickets generally have more unsaturated fatty acids (UFA) compared to saturated fatty acids (SFA) (59, 61). A notable exception occurs in *T. portentosus*, which has more SFA compared to UFA (70). This difference could be a result of unreported oleic acid, which has been reported in other crickets. The majority of the non-communicable diseases, such as type 2 *Diabetes Mellitus*, obesity, cardiovascular disease (thrombosis, atherosclerosis, and high blood pressure), and some cancers affecting human beings, are due to the consuming of SFA (22). Consumption a high PUFA and MUFA in crickets is therefore capable of reducing the detrimental effects of high-SFA diets (22).

### Mineral Composition of Different Species of Edible Crickets

Edible crickets are a good source of mineral elements such as phosphorus, sodium, potassium, calcium, magnesium, iron, and zinc. Based on dry matter, edible crickets have phosphorus ranging from 0.80 to 1169.60 mg/100 g; potassium ranging from 28.28 to 1079.90 mg/100 g, and sodium ranging from 0.99 to 452.99 mg/100 g as the most abundant mineral macro mineral elements. The differences in the macro-mineral elements could be due to the diet the crickets feed on in different parts of the globe. The differences could also be due to the age of the cricket, species, contaminants, especially heavy metal during the time of processing the crickets, and the measuring methods. In this review, the majority of the crickets have higher macro-mineral elements than those found in beef, pork, and broiler chicken, although some have similar content, and a few crickets exhibit a low content of macro-mineral elements (Table 4). Edible crickets lack a mineralized skeleton and hence have very little calcium, ranging from 4.98 to 240.17 mg/100 g dry weight; however, when we compare the calcium content of the edible crickets in this review, it is higher than that of beef, chicken, and pork (66, 118). This is because the bones that have calcium reserves do not form part of the muscle tissue that is subjected to analysis. Edible cricket species in this study possess calcium that ranges between 4.98 and 240.22 mg/100 g dry weight. *Gryllus bimaculatus* cricket contains the highest amount while *Brachytrupes sp*. contains the least amount of calcium (Table 4). This finding contradicts the finding by (122) that showed that crickets contain calcium of 33–341 mg/100 g dry matter. The sodium level in edible crickets is higher compared to the one recorded in other Orthopterans and other edible insects (5, 66, 107). The micro-mineral elements such as zinc, manganese, iron, copper, cobalt and aluminum in edible crickets are higher in content compared to micro-mineral elements in beef, chicken, and pork (123, 124) (Table 4). Most of the edible crickets have higher iron content than livestock meats, although we currently have scant information concerning the iron bioavailability of crickets (125, 126). A rare study of iron bioavailability found that consuming the *G. bimaculatus* cricket can enable you to meet a high percentage of your recommended daily iron intake (127). In this case, a child must consume 120.08 mg of the G. bimaculatus to acquire the 11.60 mg/100 g recommended daily iron intake. An adult human is supposed to consume 283.64 mg of cricket to meet the recommended daily intake of iron (27.40 mg/100 g).

**Table 4:** Mineral-Nutrient elements composition (mg/100 g DM) of different species of edible crickets and selected animal tissues (mg/100 g DM).

**Cricket species**	**Mineral element**											
	**Phosphorus**	**Potassium**	**Sodium**	**Calcium**	**Magnesium**	**Zinc**	**Manganese**	**Iron**	**Copper**	**Cobolt**	**Aluminum**	**References**
*Gryllus bimaculatus*	1169.60	1079.90	452.99	240.17	143.65	22.43	10.36	9.66	4.55	NR	NR	(66)
*Teleogryllus ema*	1085.4	895.50	278.23	193.54	152.48	18.47	5.86	10.75	2.19	NR	NR	(66)
*Acheta domesticus*	832.9	126.62	435.06	171.07	94.71	20.22	3.35	8.75	1.43	NR	NR	(105)
*Tarbinskiellus portentosus*	506.1 ± 2.33	1240.89 ± 1.05	370.81 ± 0.82	26.00 ± 0.02	10.50 ± 0.06	7.0 ± 0.00	NR	122.5 ± 0.00	4.50 ± 0.01	NR	NR	(70)
*Brachytrupes membranaceus*	126.9	NR	NR	9.21	0.13	NR	NR	0.68	NR	NR	NR	(116)
*Brachytrupes spp*	38.50 ± 1.91	877.26 ± 41.39	150.22 ± 28.23	4.98 ± 0.58	NR	23.02 ± 0.06	NR	33.60 ± 3.26	NR	NR	NR	(66, 67)
*Gymnogryllus lucens*	NR	28.28 ± 17.88	15.63 ± 5.30	NR	153.88 ± 27.47	25.66± 28.70	NR	51.90 ± 44.5	6.91 ± 0.70	0.21 ± 0.70	NR	(117)
*Gryllus assimilis*	0.80	NR	0.99	45.30 ± 4.45	2.19 ± 3.46	5.22 ± 0.27	1.42 ± 0.09	2.78 ± 0.28	0.68 ± 0.01	NR	4.21 ± 2.51	(63, 118)
**Animal tissue**												
Beef	NR	NR	NR	5.43	49.33	5.53	0.04	3.31	0.45	NR	NR	(76)
Broiler chicken	407.00	248.00	46.00	5.80	29.00	0.70–1.30	NR	0.40–0.70	0.04–0.10	NR	NR	(107)
Pork	223.00–320.00	370.00–400.00	45.00–87.00	4.30–6.00	21.00–26.10	2.40–6.90	NR	1.40–21.00	0.10–2.70	NR	NR	(107)
**Recommended nutrient intake (mg/day)**												
Children	100.00			300.00	26.00	2.80	0.003	11.60				(77, 119–121)
Adults	700.00	2000.00	500.00	1300.00	260.00	7.20	2.30	27.40	1.50			(77, 119–121)

*NR, Not Reported*.

### Vitamins Composition of Different Species of Edible Crickets

Edible crickets are an excellent source of a wide range of water-soluble vitamins and lipophilic vitamins, including thiamine, riboflavin, niacin, and vitamin B12 (62, 105) (Table 5). House cricket, *A. domesticus*, contains 0.4 mg of thiamine per 100 g of dry weight, which is within the range of 0.1 to 4 mg per 100 g of dry matter thiamine content reported in other edible insects (72). Riboflavin in edible crickets ranges from 0.23 to 3.41 mg/100 g. *Gryllus assimilis* cricket is richer in vitamin B12 with a content of 5 mg per 100 g compared to *A. dometicus* (62). Retinol (vitamin A) and β-carotene were detected in *A. domesticus*, while only Retinol was detected in *G. assimilis. A. domesticus* possess a retinol content of up to 67 g/100 g dry weight and a β-carotene of up to 0.02 g/100 g dry weight (105). *Gryllus assimilis*, on the other hand, has a retinol content of 2.90 mg/100 g of dry matter (62). Vitamin E is found in both *A. domesticus and G. assimilis* (Table 5). This review shows that the edible crickets are a good source of riboflavin, pantothenic acid, biotin, vitamin A, vitamin C, niacin, and thiamine. This is in line with the findings of Rumpold and Schlüter (5), who reported that insects consumed as food and feed are usually rich in riboflavin, pantothenic acid, and biotin. On the other hand, however, they are poor sources of vitamin A, vitamin C, niacin, and in most cases thiamine. The number of vitamins in edible insects collected from the wild is seasonal and influenced by the meal the insect consumes. This problem of seasonal availability of the vitamin can be overcome through the rearing of the crickets on farms using diets rich in vitamins. The review shows that edible crickets can meet the recommended daily intake of most of the vitamins. This can be achieved by either increasing the number of mg/ 100 g for those vitamins that are in low supply in crickets or by reducing the mg/100 g consumed where the vitamins contained in the edible cricket is high.

**Table 5:** Vitamin composition of different species of edible crickets.

**Vitamin**	**Cricket species**		**Recommended daily intake**		
	***Acheta domesticus***	***Gryllus assimilis***	**Children**	**Adult female**	**Adult male**
	**mg/100 g**	**mg/100 g**	**mg/day**	**mg/day**	**mg/day**
Retinol (Vitamin A)	<67.00	2.90 ± 0.05	6	15	15
β carotene	<0.02	NR	NR	NR	NR
Thiamine (Vitamin B1)	0.04	NR	0.4	1.1	1.2
Riboflavin (Vitamin B2)	3.41	0.23 ± 0.08	0.3	1.1	1.3
Niacin (Vitamin B3)	3.84	NR	2	14	16
Pantothenic acid (Vitamin B5)	2.30	NR	1.7	5	5
Pyridoxine (Vitamin B6)	0.23	NR	0.0001	0.0013	0.0013
Biotin (Vitamin B7)	0.02	NR	0.005	0.03	0.03
Folic acid (Vitamin B9)	0.15	NR	0.65	0.40	0.40
Vitamin B_12_	0.01	10.00 ± 0.00	0.004	0.0024	0.0024
Vitamin C	3.00	1.01 ± 0.63	15	65	75
Vitamin D	<17.15	NR	5	5	5
Vitamin E	1.32	30.00 ± 0.01	6	15	15
Vitamin K	NR	40.00 ± 0.00	0.03	0.065	0.065
Choline	151.90	NR	125	425	550
References	(105)	(62)	(128)	(121, 128)	(121, 128)

*NR, Not Reported*.

## Sensory Qualities of Edible Crickets

Crickets captured from the wild or the one raised in the farms must be processed before they are consumed. During processing, the crickets are starved for 1–3 days before they are killed humanely by scalding them using hot water (129). After killing them, they are then cooked, smoked, fried, toasted, dried, or processed into cricket products, such as crackers, to improve their taste and palatability (50, 129, 130). The method of preparation of crickets and their products play an important role in influencing a person's willingness to sample and consume them. Before consumption, the consumer will employ their sensory organs, such as smell, touch, sight, and sound, to choose whether or they will eat it.

Sensory attributes as they relate to the processed crickets and their products therefore influence cricket consumption. Processed crickets and their products have diverse taste, color, and flavor. The flavor of the cricket depends on their surface odor (130). The flavor of crickets also depends on the diet they eat. Diet choices for crickets can also be adapted depending on how we want to the crickets to tase. During cooking, crickets tend to take the flavor of the additives.

The exoskeleton of crickets has a high impact on the texture of the cricket, i.e., its crunchiness. Crunchy crickets or their products tend to produce an accompanying sound like that of crackers or pretzels while being eaten (130). Nymphs of about 6–7 weeks are the stages of crickets when they are most consumed, as they contain a low quantity of chitin. The reductions of the chitin make these crickets less crispy during their consumption and increase their digestibility. A pleasant color does not always indicate the deliciousness of the cricket but only influence the consumer to accept the cricket. During cooking, the cricket color may change from the initial shades of gray or brown to red, or its color may be retained, especially if the cricket is black (130). Crickets containing a considerable amount of oxidized fat, or improperly dried crickets, may be black, which is a color that may discourage consumers. Properly dried crickets are golden or brown and can be easily crushed by the fingers (129).

## Other Benefits Derived from Crickets

Crickets possess benefits other than being consumed as food by human beings. These benefits include the following.

### Crickets as a Source of Medicine

Humans have used crickets and their products for therapeutic functions since ancient times (47, 131–134). Recent studies have shown that crickets can be utilized as a traditional remedy for fever and high blood pressure (135). The cricket legs are ground into a powder and mixed with water and then taken as a drink to relieve dropsy (oedema) (134, 136–138). In Nigeria, the intestinal content of mole crickets (*Gryllotalpa africana* Beauvois) is applied to patients suffering from athlete's foot for treatment (134, 139). In some places, *Brachytrupes* sp. crickets are also consumed as food supplements for healthy mental development and pre- and pro-natal care purposes (134, 140). In China, edible Chinese mole crickets are sun-dried to make a herb called China Gryllotalpa, which is then used as a decoction or is made into a tincture to enhance bodily functions (141).

Research has been conducted on the utilization of crickets as a new supplementary diet to fight deficiency diseases, such as Marasmus and Kwashiorkor, in schoolchildren (36, 142). The findings are interesting in that the incorporation of cricket powder in diets of the schools optimized the growth and learning of the children (142). Moreover, the presence of essential amino acids, including valine, lysine, threonine, and methionine, in edible crickets help in breaking down of saturated fatty acids, which are implicated in lifestyle conditions like obesity, hypertension, type 2 diabetes, and cancer in human beings (143). Previous studies have also shown that the cricket powder is rich in most of the mineral-nutrient elements deficient in human beings, such as calcium, potassium, magnesium, iron, and copper. One can thus obtain these minerals that are important in fighting various diseases, such as osteoporosis, malfunction of the nervous system, and anemia, by consuming the edible crickets. Direct consumption of crickets has also been shown to decrease ethanol levels in the blood by the help of enzymes such as alcohol dehydrogenase (ADH) and acetaldehyde dehydrogenase (ALDH), which stimulates the liver mitochondria to break down alcohol that may damage the liver (144). Glycosaminoglycan (GAG), which mediates anti-atherosclerotic and antilipemic effects, have been confirmed in crickets, and one can attain this compound by consuming the crickets (145). Besides, cricket extracts have been studied as a therapeutic agent for inflammatory diseases, such as chronic arthritis and gut inflammation (95, 135, 144).

### Crickets as Livestock Diets

The high nutritional content of edible crickets, the small space requirement for their production, and the effect they have on the environment make them valuable as animal feed. Moreover, crickets have an added advantage since they have already been in use as an ingredient in animal feed (146). Crickets can be given to the animals as feed either whole after killing them or can be crushed into a powder and then used to formulate livestock diets. Livestock feeds formulated by incorporating crickets are cheaper compared to the cost of commercial feeds, which currently account for 70% of the cost of livestock production (147). The most promising crickets for production of livestock feed are *A. domesticus, G. veletis, G. bimaculatus, G. sigillatus, T. mitratus, G. mitratus, T. emma, B. portentosus*, and *G. assimilis* (50, 146, 148–152). Recent studies have shown that cricket meal can partially replace broiler mash, especially the protein part. Cricket meal can replace 5–15% of fish meal or soy meal without any negative effects on broiler feed intake, weight gain, or feed conversion efficiency (153). Also, replacing the protein composition with *Gryllus testaceus* cricket meal in a broiler chicken diet did increase the essential amino acids in the chicken (150). In addition to the nutritional value, the insect-based feed could have a further advantage in improving the taste of final meat products (154).

Another report has demonstrated that African catfish (*Clarias gariepinus*) diet containing 100, 75, 50, 25, and 0% fish meal can be successfully changed to contain 0, 25, 50, 75, and 100% *G. bimaculatus* crickets, respectively (151). Furthermore, growth performance and body composition could be improved when African catfish were provided with a diet containing 50–100% crickets (151, 152). As compared with commercial fish-based meals, a cricket-based meal significantly increased the body weight, resistance to diseases, protein efficiency ratio, and specific growth rate of the catfish (152). The study also found the fish provided with 100% cricket meal contained a significantly lower feed conversion ratio compared to the lower inclusion level. The findings further revealed the whole-body crude protein value was higher in catfish fed with a meal containing 50–100% cricket meal.

In summary, existing studies have clearly demonstrated that crickets are a promising protein source for animal meals and can meet the increasing global requirement (5). Before the mass-production of such cricket-based meals, however, governments and companies should ensure health and safety concerns relating to edible crickets, such as the presence of anti-nutrient properties and legislation of use of these crickets, are addressed (57).

### Cricket Harvesting as a Strategy of Pest Control

In recent years, there has been a prevalence of crickets in warm areas around the world, which has caused a remarkable loss of field crops and other plants. Moreover, when the crickets get into domestic houses they become a significant nuisance by destroying household properties. The capturing and consumption of edible crickets therefore not only ensures nutritional availability to people and livestock but also protects the plants and household properties from unexpected insects infestation (http://www.entomoljournal.com/archives/2016/vol4issue6/PartA/4-5-101-553.pdf; https://www.bbc.com/news/av/world-middle-east-52991180/pakistan-locust-plague-locals-collect-insects-for-chicken-feed). Gathering crickets from farms and consuming them as food can also help in reducing pesticide use in controlling these cricket pests. This, in return, can protect the environment from pollution, minimizing the killing of other useful insects and poisoning of consumers (78). An excellent example of a place where the gathering of crickets for human consumption or feeding chickens is Mexico, where this has contributed to a reduction in the cricket population of farms, a reduction in the amount of pesticides used in crops, and a decreased financial burden on farmers (155, 156).

### Cricket Contribution to Economic Development

Collection and rearing of crickets provide employment and cash income to people both at the household level and at the level of industrial production. For example, in Africa, Asia, and Latin America, there is a demand for edible crickets, and this makes it easier to bring them into the market for sale (157–159). The crickets are sold at local markets while alive or after being killed. Live crickets are packed into various weights for various buyers and then brought to the streets of towns and the markets for sale. Alternatively, the crickets are killed and are either fried or are processed into cricket products as human food or chicken and fish feed and then brought to the market for sale (32–34, 159, 160). The cricket businesses that occur in many countries are usually influenced by the demand of the local people or immigrant communities because of the development of a specific market that sells cricket products. Cricket collection and farming have also led to further opening up for international trade, such as border trade in edible crickets, which is commonly practiced in the Southeast part of Asia and Central Africa (159).

### Crickets and Livelihood Improvement

Edible crickets trapped from the wild or domesticated in farms play a role in livelihood improvement by providing an improved diet in terms of nutritional content and diversity and as a supplement to the dietary needs of low-income families. These crickets also provide food at times of famine for people living in developing countries and Western countries. The resource-poor people in the society, including women and landless people living in urban centers and rural areas, can actively involve themselves in the collection, rearing, processing, and selling of the surplus of crickets and their products in the streets of towns. These ventures can significantly change their quality of life through the generation of income, which, in return, they can use to purchase the basic needs they are lacking. Crickets could be directly and easily gathered from the wild or reared on the farm with a little technology and by involving much capital in procuring basic rearing and harvesting equipment (161). Rearing crickets requires a small portion of land and minimal market introduction efforts, as crickets already form part of some local food cultures (32, 162). Malnutrition is a widespread issue affecting many disadvantaged members of society, especially during times of social conflict and natural disasters. Since crickets are nutritionally rich and easily accessible, having simple rearing techniques and rapid growth rates, they can offer a cheap and efficient chance to mitigate food insecurity. The edible crickets and their products can be provided for hunger-stricken people as a relief food and thus improve their livelihoods. Furthermore, cricket flour and powder can be used to fortify the traditional food in different communities before feeding vulnerable persons in society to improve their livelihood (36, 142).

### Crickets as a Food Preservative

Chitosan from the chitin of the edible cricket species exoskeleton has been identified to be a possible intelligent and biodegradable bio-based polymeric material for packaging of various foods. Such natural packaging using the “exoskeleton” of crickets can change the internal conditions of the food product, thereby protecting the food product from spoiling and micro-organisms. This is possible because it has been proved that chitosan from crickets stores antioxidants and has antimicrobial activity against yeasts, molds, and bacteria (163–165). However, the chitosan polymeric material can be compromised when it gets into contact with moisture and may therefore not be utilized in true natural form but may be synthesized further into chitosan film for a positive impact to be achieved (165).

### Singing Crickets as a Source of Music

Rearing of crickets as pets has existed since prehistoric time in Asia some Western countries. For instance, crickets have been mentioned in an adage dating back to 600 BCE found in Ancient Greece: a young girl and her dying pet cricket. Since then, some poetry work has been scripted on the songs of crickets (166). In the People's Republic of China, singing crickets have been household pets for more than 2,000 years. During the Tang Dynasty (618–906 CE), Chinese people reared crickets in small cages for their songs. Whenever the autumn arrived, the ladies of the palace trapped crickets and placed them in small golden cages, which they placed near their pillows to listen to their songs when night fell. This tradition was also embraced by ordinary people (167). Some South American crickets have been implicated to have beautiful songs that made Amerindians people rear them (168, 169). In the Luo community in Kenya, there is a traditional belief that eating crickets improves the vocal prowess of musicians. As such, during music festivals, children who are members of the school choir would hunt and eat crickets to improve their vocal ability (32).

### Cricket Fighting as a Sport

Edible crickets can be used as sporting activity for recreation purposes. In China, Cricket fighting has existed from the time of the Song Dynasty (960–1278 CE). This practice of allowing crickets to fight was later declared illegal during the Qing Dynasty (1644–1911 CE). Currently, however, cricket fighting is legal, and it has become a common sporting activity in many Chinese cities, such as Beijing, Guangzhou, Huwan, Hong Kong, Shanghai, and Tianjin, where cricket fighting clubs and societies have been formed (170). Cricket fighting has spread to other parts of the world, such as New York and Philadelphia (171) as a result of the migration of Chinese people to these places. During the sport, people gather together in social halls with their crickets to get entertained as the crickets fight. The best example of the fighting cricket is the Chinese fighting cricket *T. mitratus*.

### Promotion of Cultural Entomology

Crickets have contributed a lot to the shaping of literature, art, and doctrine in societies around the world—referred to as cultural entomology (172). Contributions from this discipline have assisted in highlighting the different roles the crickets have undertaken in literature, especially in children's books, movies, and visual art, as collected artifacts, decoration, and especially as inspiration for innovative expression.

The crickets have also played different roles in folklore and superstition in different parts of the globe. In this perspective, some communities hold a lot of esteem for crickets since they believe that once you hear the song of the cricket it spells good fortune, although others say it is a bad omen when a cricket makes noise around you. In China, for instance, the crickets have been implicated to foretell the coming of rain, death, or the returning of a lover who has been away (173). Moreover, the people of China keep crickets in a small cage to have good luck (174). In Barbados, when a singing cricket enters the house, it spells the fortune of money into the family, and no person is therefore allowed to kill or chase away the visiting cricket. On the other hand, when a quiet or less noisy cricket gets into the house, it forecasts sickness or a pending death in the family (175). Omens concerning crickets are also found in Brazilian folklore where they bear different meanings to various events. For instance, when a black cricket gets into a house of someone, it indicates that a person in that house will be sick while a gray cricket is a sign of money coming to the household (176). A cricket also foretells the pending death of a member of the family, and, therefore, whenever a cricket sings in the house, it is captured and killed immediately to avert the death (177). In the other parts of Brazil, the cricket spells the pregnancy in a member of the household when it sings non-stop. If it sings and breaks, it then spells a windfall of money to that home (178). A singing cricket also directs people to the source of drinking water during droughts. In the case of a cricket aboard a sailing ship, singing foretells the proximity of land to the captain and sailors (176).

A mole cricket (genera *Scapteriscus* and *Neocurtilla*, Gryllotalpidae) that enters into the house of a Brazilian brings both good luck and rainfall (178). When it digs tunnels in the soil, loosening it, people usually interpret this behavior as a sign that rainfall is imminent (178). It is said that the appearance of a mole cricket on the surface of the ground is an indicator that the soils are waterlogged after a heavy downpour or they are ready to disperse to occupy new areas (179). In Zambia, there is a belief that whoever comes across an African mole cricket will have luck (180). Zambians therefore keep mole crickets to retain luck.

## Risks of Cricket Consumption

Consumption of crickets is generally safe. However, it could expose one to various risks that must be taken into consideration. For instance, (44) has published various risk profiles related to the consumption of crickets. Gathering a large number of crickets from the wild for consumption or sale could cause a serious imbalance to the ecosystem (181). To overcome such an effect on biodiversity, it is advisable to rear crickets at a farm level under controlled and defined conditions for consumption for food and marketing. This means that farm rearing of crickets must be done with appropriate and safe substrates to guarantee the health and safety of consumers. The wrong choice of cricket diet may pose a health hazard to consumers. For instance, the result of analyses carried out from 2003 to 2010 indicated possible risks of consuming heavy metals due to the nature of the bran used as a substrate (182). Additionally, consuming crickets reared in inappropriate organic waste is discouraged. Furthermore, some crickets can also contain naturally poisonous compounds such as cyanogenic glycosides (183). Cyanogenic glycosides are natural plant toxins that are present in several plants, most of which are consumed by crickets.

Consumption of crickets containing cyanogenic glycosides may cause acute poisoning, leading to growth retardation and neurological symptoms due to a damaged central nervous system (CNS) (184). The other likely risks of consuming edible crickets include poor handling and delish treatment. According to (185), consuming crickets with their feet can cause intestinal discomfort based on the amount ingested. Eating crickets can also cause allergies to those persons sensitive to insect chitins. Some individuals have such a small amount of chitosan enzyme that the eating of crickets can cause an allergic reaction to them (44). Some crickets have a tough exoskeleton formed of chitin, which is difficult to digest for humans.

The risk of contracting zoonotic diseases from some cricket species must also be taken into consideration. The intestinal flora of crickets could be a predisposing agent for the growth of unwanted microorganisms. Klunder et al. (86) evaluated the microbial content of fresh, processed, and stored edible crickets *A. domesticus* and *Brachytrupes*. The results showed that various types of Enterobacteriaceae and sporulating bacteria can be identified and subsequently seperated from raw crickets entering them most likely during contact with the soil (186). Fasting, heat treatment, and appropriate storage conditions are paramount to dangerous disease-causing pathogens in crickets and other edible insects (155, 187).

## Cricket Farming Around the World

The high potential of crickets as food and feed has led to the development of rearing systems and establishment of subsistence and commercial cricket farms in several countries in Asia, Europe, America, Australia, and, recently, Africa. Asia is the leading continent in cricket farming in countries such as Thailand, Indonesia, Cambodia, Myanmar, and Lao Peoples' Democratic Republic (PDR) (35, 188, 189). Examples of edible cricket species that have been farmed successfully in Asia for food and feed include the house cricket *A. domesticus, G. bimaculatus, T. occipitalis, T. mitratus, G. testaceus*, Jerman cricket (*Gryllus* sp.), the short-tail cricket *B. portentosus*, and *T. portentosus* (35, 54, 146, 189–191). *Acheta domesticus* is the preferred cricket species for large-scale production for most parts of the globe (192).

Cricket farming in Thailand, which is said to be the hot spot of cricket consumption, has an established cricket industry with over 20,000 farms producing cricket products such as adult crickets, eggs, and biofertilizer from cricket waste for commercial purposes (35, 146, 193, 194). Farmers in Thailand rear two cricket species: *A. domesticus, G. bimaculatus*. They, however, prefer rearing *G. bimaculatus*, which form a greater portion of the Thai production since *G. bimaculatus* has a short lifecycle and is stronger and hardy, though less popular than *A, domestica* (146). Thai farmers initially used to rear their crickets in small concrete cylinders, plastic boxes, wood, and other types of containers, but of late they farm crickets in large pens with concrete walls (193). These pens are easy to clean, cheap to build, and durable. Several egg trays supply the living section of the pen as hiding places for the crickets. Predators that may kill the crickets are kept off the rearing pens and the farm by use of mosquito nets. The cricket eggs used to start a colon are either purchased or tapped from crickets in the wild by supplying bowls containing clean egg-laying substrates, such as rice bran, wheat bran, ash, or fine sand soil, for the females to lay the eggs. Eggs take 7 to 10 days to hatch. Each harvesting cycle is between 28 and 35 days. Cricket farming in Thailand follows a family-owned business model which produces about 3,000–7,000 tons of crickets per year (49). A medium-scale farm can yield 500–750 kg of mature crickets per 45-days harvesting cycle, which generates a revenue of about 2,000–2,500 USD (193).

In Indonesia, cricket farming is extensively practiced in several cities of the Java islands for livestock feed, home consumption, and business purposes. The crickets are farmed in Java cities, including Cirebon, Bekasi Demak, Kudus, Purwodadi, and Yogyakarta and in East Java in Tulungagung, Kediri, and Porong (54). Cricket production in Cirebon is 200 kg of young crickets and 8 kg eggs per day. However, some small-scale cricket farmers have been reported in some villages, where farmers rear crickets to feed their poultry or as an ingredient for medicines (149). The crickets farmed in Indonesia are *G. bimaculatus, G. testaceus, G. mitratus*, and the German cricket *Gryllus* sp.

Cricket farming has just been initiated in South Korea with the support of the Korean government, which has already established legal measures to support the cricket industry. Currently, several research projects are being carried out in South Korea under the guidance of the Rural Development Administration. This has led to an increased number of *G. bimaculatus* farms in South Korea (195). This has further led to a Korean company using an automated farming system for large-scale production of the crickets (196).

In Cambodia, cricket farming is of small-scale production aimed for home consumption (193). This is as a result of the farmers in Cambodia being resource poor. They therefore rear crickets in small farms using small plastic containers, wood, and other types of containers. The Cambodian farmers use plastic bags instead of egg trays as a living area for the crickets. Whereas, these bags are said to be cheaper, they pose a risk to the crickets which will consume the particles of the bags. Farmers use ash in the water containers to avoid small crickets drowning. It is unclear if this is a good method. Sometimes farmers spraying small particles of water on the floor of the rearing pens for the crickets to drink but this may be safe for the crickets since the water may generate too much humidity in the rearing pen. Recently, some farmers are enrolled in training programs to get equipped with better information on how to rear healthy crickets. There are breeders of crickets in Myanmar, but no farmers have been reported. Likewise, there are a few cricket farmers in Malaysia (197). The Philippines collect their edible crickets from the wild (193, 198).

Cricket farming in western countries is a new trend that is about 10 years old. The house cricket (*A. domesticus*), brown field cricket (*G. assimilis*), and two-spotted field cricket (*G. bimaculatus*) are the common crickets bred in Europe and for industrial processing (44, 148). The western follows a farming model that aims at optimizing breeding activities by reducing human labor during the production of the crickets. Their model aims at raising crickets on a large-scale basis for industrial processing unlike for the Asian model that aims at producing enough for subsistence use. Due to the tough conditions of EU regulations, there are a few farms rearing crickets for food in Europe in countries such as Belgium, France, Finland, and the Netherlands (44). The largest cricket farms for food in the EU are run by a company called Kreka, which is based in the Netherlands. In Finland, the Nordic Insect Economy used to be a major cricket producer, but production as decreased (193). Finland is also the home of Entocube, a startup that began by rearing crickets in containers placed in urban areas but which has now progressed to a new 250,000 Euro project of farming crickets in a 60-years-old mine, taking advantage of the 28°C temperatures emanating from the geothermal station. Cricket farming in North America is practiced in Canada and the US. In Canada, cricket farming is carried out by private companies such as Entomo Farms, Third Millenium, and next Millenium. These companies rear crickets to sell as dry insects and/or processed cricket powder and flour to most of the edible cricket startups in the US, where there are only a few cricket farms (199, 200). In the USA, cricket farming is undertaken by Aspire Food Group, which started its cricket flour processing with the Aketta brand, has expanded its market activities by purchasing the cricket energy bar brand Exo in 2018. The cricket farmed in the US is *A. domesticus*.

In Africa, cricket farming is at its infancy stage in countries such as Kenya, Uganda, Mali, and Madagascar (32, 201, 202). Cricket-rearing technology has been disseminated to farmers in these countries through mass media (televisions, radios, and print materials) and training of students and farmers. The institutions of training include Jaramogi Oginga Odinga University of Science and Technology, Jomo Kenyatta University of Agriculture and Technology and Makerere universities, and the International Center for Insect Physiology and Ecology (*icipe*). A grant from the Danish government facilitates the technology transfer of cricket farming through GREENiNSECT project that supports “the rearing and eating crickets as a delicious, affordable and healthy solution for malnutrition.” The project has accelerated, leading to the establishment of small-scale cricket farms in L. Victoria region, which was the initial point of introduction in Kenya and Uganda. From this point of introduction, cricket farming has spread to other regions, such as central Kenya and the coast of Kenya. Three cricket species, *S. icipe, A. domesticus*, and *G. bimaculatus* are reared (28, 29, 203, 204). In most cases, these cricket species are reared in the same farm by one farmer; however, in some instances, they are reared in separate farms by different farmers. The cricket species reared by the farmer depends on which species is more appealing to him or her. *Scapsipedus icipe* and *A. domesticus* are most popular among farmers because they are softer than *G. bimaculatus*. In Kenya, there are about 300 cricket farmers who produce 28,800 kg of crickets per year (205). The cricket farm capacity can produce about 160 kg of crickets/ one harvesting cycle of 60 days. According to the field survey by Ayieko et al. (32), the largest production volume of farmed crickets is at Bondo and Kisumu counties in the Nyanza region. Most farmers use rectangular plastic containers while others rear the crickets in cylindrical plastic containers that have ventilation covered with plastic netting (28, 29),. The yield is low at 4 kg per cage at the harvesting stage.

Farming of crickets requires varying degrees of labor input during the rearing cycle (29). Each day one person is involved in the transfer of the egg containers from the main enclosures to the empty egg-laying enclosures and for daily feeding of crickets. This requires one person for 1 h of work. But where large-scale farming of crickets is practiced, such as in Thailand, more people are required to work in the cricket farms (146). Cricket farming in Kenya has picked up since cricket requires a small starting and maintenance capital and it is easy to set up farms for crickets. Farmers must explore the idea of adding value to their crickets by processing them. Moreover, rearing of crickets will ensure there are enough crickets for consumption and to stop depending on wild-collected crickets.

## Conclusion

The current study has shown that consuming crickets as food by human beings is traditionally practiced in 49 countries around the world. Over 60 cricket species are known to be edible. Crickets are a highly nutritious food resource and may therefore be included in the list of the common diet of global consumers in the future. These crickets could also be used as nutritional supplements for special diets for schoolchildren, sick people, and athletes. Inclusion of potentially suitable species of crickets into the normal diet requires defined and standardized conditions of their rearing as well as the detailed monitoring of their composition, including biologically active compounds. Though the EFSA and *icipe* have already assessed hygienic and toxicological and microbial risks related to edible crickets, more research on their composition and nutrient profile should be carried out to fully implement edible crickets as food into the global legislation documents. Currently, there are only a few cricket species that are farmed. The farmers must be encouraged to start rearing other species of crickets that have not yet been confined. Also, animal breeders should try to find out whether it is possible to crossbreed the crickets with a long lifecycle with the ones with a short lifecycle.

## Contribution to the Field

Edible crickets have become popular in the past few years not only in the scientific literature but in other platforms as well. One of the major advantages of eating crickets is their impressive nutritional composition. Many sources report that crickets have better nutritional characteristics than traditional protein sources. In our research, we aimed to give a complete picture of edible crickets in the world, their nutritional profile and other benefits. The materials we used are published results of different authors from the past few years. The list of crickets provided by various authors' shows that there are 66 crickets that are consumed as food and feed in the world and crickets generally have a better nutritional profile than other meats. Based on our findings, crickets have a promising nutritional profile in terms of energy, protein, lipids and important fatty acids, mineral elements vitamins, carbohydrate and medicinal elements and may become part of many food products in future. As an enterprise, cricket farming, can mitigate food insecurity, act as a source of income when sold and a source of employment. The present review provides comprehensive information on the diversity of crickets, their nutritional values and their potential to contribute to the livelihood of mankind.

## Author Contributions

HM, SN, MA, MM, SE, JE, EK, JO, SH, KF, MO, NR, and CT: conceptualization, writing—original draft, and writing—review and editing. HM, SN, EK, and SH: data curation. HM, SN, EK, SE, and MM: formal analysis. HM, SN, MA, MM, SH, NR, SE, JE, EK, and KF: methodology. HM, SN, and EK: software. SN, MM, JE, SE, SH, CT, and KF: validation. HM: investigation. All authors contributed to the article and approved the submitted version.

## Conflict of Interest

The authors declare that the research was conducted in the absence of any commercial or financial relationships that could be construed as a potential conflict of interest.

## References

[1] United Nations World Population Prospects 2019. (2019) Available online at: https://population.un.org/wpp/ (accessed September 15, 2020).

[2] Belluco S Losasso C Maggioletti M Alonzi CC Paoletti MG Ricci A Edible insects in a food safety and nutritional perspective: a critical review. Compr Rev Food Sci Food Saf. (2013) 12:296–313. 10.1111/1541-4337.12014

[3] Food Security Information Network 2019 Global Report on Food Crises: Joint Analysis for Better Decisions. Rome; Washington, DC: Food and Agriculture Organization (FAO); World Food Programme (WFP); and International Food Policy Research Institute (IFPRI) (2019). Available online at: https://www.fsinplatform.org/report/global-report-food-crisis-2019/ (accessed September 1, 2020).

[4] Ramos-Elorduy J Anthropo-entomophagy: cultures, evolution and sustainability. Entomol Res. (2009) 39:271–88. 10.1111/j.1748-5967.2009.00238.x

[5] Rumpold BA Schlüter OK. Nutritional composition and safety aspects of edible insects. Mol Nutr Food Res. (2013) 57:802–23. 10.1002/mnfr.201200735 23471778

[6] Evans J Alemu MH Flore R Frøst MB Halloran A Jensen AB “Entomophagy:” an evolving terminology in need of review. J Insects Food Feed. (2015) 1:293–305. 10.3920/JIFF2015.0074

[7] Van Huis A Edible insects contributing to food security? Food Secur. (2015) 4:20 10.1186/s40066-015-0041-5

[8] Bodenheimer FS Insects as Human Food: a Chapter of the Ecology of Man. The Hague: Springer (2013). p. 352.

[9] Jongema Y List of Edible Insects of the World. Wageningen: Laboratory of Entomology, Wageningen University (2017).

[10] Kelemu S Niassy S Torto B Fiaboe K Affognon H Tonnang H African edible insects for food and feed: inventory, diversity, commonalities and contribution to food security. JIFF. (2015) 1:103–19. 10.3920/JIFF2014.0016

[11] Tchibozo S Van Huis A Paoletti MG Notes on edible insects of South Benin: a source of protein. In: Paoletti MG, editor. Ecological Implications of Minilivestock: Role of Rodents, Frogs, Snails, and Insects for Sustainable Development Science Publishers. Enfield: MT (2005). p. 245–51.

[12] Lesnik JJ. Termites in the hominin diet: a meta-analysis of termite genera, species and castes as a dietary supplement for South African robust australopithecines. J Hum Evol. (2014) 71:94–104. 10.1016/j.jhevol.2013.07.015 24613098

[13] van Huis A Insects as food in sub-Saharan Africa. Insect Sci Appl. (2003) 23:163–85. 10.1017/S1742758400023572

[14] Feng Y Chen XM Zhao M He Z. Sun L Wang Y. Edible insects in China: utilization and prospects. Insect Sci. (2017) 25:184–98. 10.1111/1744-7917.12449 28225201

[15] Fasoranti JO Ajiboye DO Some edible insects of Kwara state, Nigeria. Am Entomol. (1993) 39:113–6. 10.1093/ae/39.2.113

[16] Riggi L Veronesi M Verspoor R MacFarlane C Tchibozo S Exploring Entomophagy in Northern Benin-Practices, Perceptions and Possibilities. Benin Bugs Report. Bugsforlife: London (2013).

[17] Agbidye FS Ofuya TI Akindele SO Marketability and nutritional qualities of some edible forest insects in Benue State, Nigeria. Pak J Nutr. (2009) 8:917–22. 10.3923/pjn.2009.917.922

[18] Weaving A Insects: A Review of Insect Life in Rhodesia. Regal Publishers. Irwin Press Ltd: Salisbury (1973).

[19] Mbata KJ Traditional use of arthropods in Zambia. I the food insects. Food Insects Newslett. (1995) 8:5–7.

[20] Harris WV Some notes on insects as food. Tanganyika Notes Rec. (1940) 9:45–8.

[21] Mbata KJ Chidumayo EN Lwatula CM Traditional regulation of edible caterpillar exploitation in the Kopa area of Mpika district in northern Zambia. J Insect Conserv. (2002) 6:115–30. 10.1023/A:1020953030648

[22] Kaviani S Taylor CM Stevenson JL Cooper JA Paton CM. A 7-day high-PUFA diet reduces angiopoietin-like protein 3 and 8 responses and postprandial triglyceride levels in healthy females but not males: a randomized control trial. BMC Nutr. (2019) 5:1. 10.1186/s40795-018-0262-7 32153916 PMC7050740

[23] Chakravorty J Ghosh S Meyer-Rochow VB. Practices of entomophagy and entomotherapy by members of the Nyishi and Galo tribes, two ethnic groups of the state of Arunachal Pradesh (North-East India). J Ethnobiol Ethnomed. (2011) 7:5. 10.1186/1746-4269-7-5 21235790 PMC3031207

[24] Singh OT Nabam S Chakravorty J Edible insects of nishi tribe of Arunachal Pradesh. Hexapoda. (2007) 14:56–60.

[25] Yhoung-Aree J Edible insects in Thailand: nutritional values and health concerns. Forest insects as food: humans bite back. In: Durst PB Johnson DV Leslie RN Shono K, editors. Food and Agriculture Organization of the United Nations, Bangkok (2010). p. 201–16.

[26] Lumsa-ad C Study on the species and the nutrition values of edible insects in upper southern Thailand. Kaen Kaset. (2001) 29:45–49.

[27] Sun-Waterhouse D Waterhouse GI You L Zhang J Liu Y Ma L. Transforming insect biomass into consumer wellness foods: a review. Food Res Int. (2016) 89:129–51. 10.1016/j.foodres.2016.10.001 28460898

[28] Magara HJ Tanga CM Ayieko MA Hugel S Mohamed SA Khamis FM. Performance of newly described native edible cricket *Scapsipedus icipe* (Orthoptera: Gryllidae) on various diets of relevance for farming. J Econ Entomol. (2019) 112:653–64. 10.1093/jee/toy397 30657915

[29] Orinda MA Effects of housing and feed on growth and technical efficiency of production of Acheta domesticus (L) AND Gryllus bimaculatus for sustainable commercial crickets production in the lake victoria region, kenya. (Doctoral dissertation, JOOST). (2018) Available online at: http://ir.jooust.ac.ke:8080/xmlui/handle/123456789/8852 (accessed on September 25, 2020).

[30] Oonincx DG Van Broekhoven S Van Huis A van Loon JJ. Feed conversion, survival and development, and composition of four insect species on diets composed of food by-products. PLoS ONE. (2015) 10:e0144601. 10.1371/journal.pone.0144601 26699129 PMC4689427

[31] Weiping Y Junna L Huaqing L Biyu Lv Nutritional Value, Food Ingredients, Chemical and Species Composition of Edible Insects in China. Web of Science™ Core Collection. London: BKCI (2017). p. 1–29.

[32] Ayieko MA Ogola HJ Ayieko IA Introducing rearing crickets (gryllids) at household levels: adoption, processing and nutritional values. JIFF. (2016) 2:203–11. 10.3920/JIFF2015.0080

[33] Halloran A Roos N Eilenberg J Cerutti A Bruun S. Life cycle assessment of edible insects for food protein: a a review. ASD. (2016) 36:57. 10.1007/s13593-016-0392-8 32010238 PMC6961468

[34] Halloran A Roos N Flore R Hanboonsong Y The development of the edible cricket industry in Thailand. JIFF. (2016) 2:91–100. 10.3920/JIFF2015.0091

[35] Hanboonsong Y Jamjanya T Durst PB Six-Legged Livestock: Edible Insect Farming, Collection and Marketing in Thailand. RAP Publication, 3. Bangkok: Regional Office for Asia and the Pacific of the Food and Agriculture Organization of the United Nations: (2013).

[36] Homann AM Ayieko MA Konyole SO Roos N Acceptability of biscuits containing 10% cricket (*Acheta domesticus*) compared to milk biscuits among 5-10-year-old Kenyan schoolchildren. JIFF. (2017) 3:95–103. 10.3920/JIFF2016.0054

[37] Tanga C Magara HJ Ayieko AM Copeland RS Khamis FM Mohamed SA. A new edible cricket species from Africa of the genus *Scapsipedus*. Zootaxa. (2018) 4486:383–92. 10.11646/zootaxa.4486.3.9 30313752

[38] Ssepuuya G Sengendo F Ndagire C Karungi J Fiaboe KKM Efitre Effect of alternative rearing substrates and temperature on growth and development of the cricket *Modicogryllus conspersus* (Schaum). JIFF. (2020).

[39] Bodenheimer FS Insects as human food. In: Insects as Human Food. Springer: Dordrecht (1951). p. 7–38. 10.1007/978-94-017-6159-8_1

[40] Gelfand M Insects. In: Diet and Tradition in an African Culture. Edinburgh: E&S. Livingstone (1971). p 163–171.

[41] Séré A Bougma A Ouilly JT Traoré M Sangaré H Lykke AM. Traditional knowledge regarding edible insects in Burkina Faso. J Ethnobiol Ethnomed. (2018) 14:1. 10.1186/s13002-018-0258-z 30217159 PMC6137937

[42] Bani G Some aspects of entomophagy in the Congo. Food Insects Newsl. (1995) 8:4–5.

[43] Nkouka E Les insectes comestibles dans lês societes d'Afrique Centrale. Muntu. (1987) 6:171–8.

[44] EFSA Scientific Committee Risk profile related to production and consumption of insects as food and feed. EFSA J. (2015) 13:4257 10.2903/j.efsa.2015.4257

[45] Frigerio J Agostinetto G Sandionigi A Mezzasalma V Berterame NM Casiraghi M. The hidden ‘plant side’ of insect novel foods: a DNA-based assessment. Food Res Int. (2020) 128:108751. 10.1016/j.foodres.2019.108751 31955731

[46] Instar Farming Farming Crickets for Food in the UK. (2020) Available online at: https://www.instarfarming.com/ (accessed September 1, 2020)

[47] Angie S Survey Reveals Our Appetite for Eating Insects. (2019) Available online at: https://www.newshub.co.nz/home/rural/2019/07/survey-reveals-our-appetite-for-eating-insects.html (accessed August 15, 2020).

[48] Boulos S Tännler A Nyström L. Nitrogen-to-protein conversion factors for edible insects on the swiss market: molitor T. A. domesticus, migratoria L. Front. Nutr. (2020) 7:89. 10.3389/fnut.2020.00089 32754611 PMC7366252

[49] Halloran A Megido RC Oloo J Weigel T Nsevolo P Francis F Comparative aspects of cricket farming in Thailand, Cambodia, Lao People's Democratic Republic, Democratic Republic of the Congo and Kenya. JIFF. (2018) 4:101–14. 10.3920/JIFF2017.0016

[50] Van Huis A Van Itterbeeck J Klunder H Mertens E Halloran A Muir G Edible Insects: Future Prospects for Food and Feed Security (No. 171). Rome: Food and Agriculture Organization of the United Nations (2013). p. 201.

[51] Miech P Berggren Å Lindberg JE Chhay T Khieu B Jansson A Growth and survival of reared Cambodian field crickets (*Teleogryllus testaceus*) fed weeds, agricultural and food industry by-products. JIFF. (2016) 2:285–92. 10.3920/JIFF2016.0028

[52] van Huis A Insects eaten in Africa (Coleoptera, Hymenoptera, Diptera, Heteroptera, Homoptera). In: Paoletti MG, editor. Ecological Implications of Minilivestock. New Hampshire, USA, Science Publishers (2005). p. 231–244.

[53] Chavunduka DM Insects as a source of protein to the African. Rhodesia Sci News. (1975) 9:217–20.

[54] Fuah AM Siregar HC Astuti DA Cricket Farming in Indonesia: Challenge and Opportunity. Bogor: LAP LAMBERT Academic Publishing (2016).

[55] Garber S The Urban Naturalist. Family Gryllidae. Toronto, ON: General publishing Company Ltd (2013). p. 61.

[56] Otte D Cigliano MM Braun Holger; Eades D.C (2018). “Infraorder Gryllidea”. Orthoptera Species File Online, Version 5.0, Facultad de Ciencias Naturales y Museo, Universidad Nacional de La Plata (UNLP).

[57] Dobermann D Swift JA Field LM Opportunities and hurdles of edible insects for food and feed. Nutr Bull. (2017) 42:293–308. 10.1111/nbu.12291

[58] Barker D Preliminary observations on nutrient composition differences between adult and pinhead crickets, *Acheta domestica*. Bull Assoc Reptil Amphib Vet. (1997) 7:10–3. 10.5818/1076-3139.7.1.10

[59] Mlček J Adámková A Adámek M Borkovcová M Bednárová M Kourimská L Selected nutritional values of field cricket (*Gryllus assimilis*) and its possible use as a human food. Indian J Tradit Know. (2018) 17:518–24. Available online at: http://nopr.niscair.res.in/handle/123456789/44581

[60] Józefiak D Józefiak A Kierończyk B Rawski M Swiatkiewicz S Długosz J 1. a review *Ann Anim Sci*. (2016) 16:297–313. 10.1515/aoas-2016-0010

[61] Tang C Yang D Liao H Sun H Liu C Wei L Edible insects as a food source: a review. Food Prod Process Nutr. (2019) 1:8 10.1186/s43014-019-0008-1

[62] Oibiokpa FI Akanya HO Jigam AA Saidu AN Nutrient and antinutrient compositions of some edible insect species in Northern Nigeria. Fountain IJONAS. (2017) 6:9–24.

[63] Araújo RRS dos Santos Benfica TAR Ferraz VP Santos EM Nutritional composition of insects *Gryllus assimilis* and *Zophobas morio*: potential foods harvested in Brazil. J Food Compos Anal. (2019) 76:22–6. 10.1016/j.jfca.2018.11.005

[64] Adámková A Mlček J Kourimská L Borkovcová M Bušina T Adámek M. Nutritional potential of selected insect species reared on the island of sumatra. Int J Environ Res Public Health. (2017) 14:521. 10.3390/ijerph14050521 28498340 PMC5451972

[65] Kourimská L Adámková A Nutritional and sensory quality of edible insects. NFS J. (2016) 4:22–6. 10.1016/j.nfs.2016.07.001

[66] Ghosh S Lee SM Jung C Meyer-Rochow VB Nutritional composition of five commercial edible insects in South Kor. J Asia Pac Entomol. (2017) 20:686–94. 10.1016/j.aspen.2017.04.003

[67] Akullo J Agea JG Obaa BB Okwee-Acai J Nakimbugwe D Nutrient composition of commonly consumed edible insects in the Lango sub-region of northern Uganda. Int Food Res J. (2018) 25:159–66.

[68] Wang D Yao YB Li JH Zhang CX Nutriotional value of the field cricket (*Gryllus testaceus* Walker). Insect Sci. (2004) 11:275–83. 10.1111/j.1744-7917.2004.tb00424.x

[69] Sánchez-Muros MJ Barroso FG Manzano-Agugliaro F Insect meal as renewable source of food for animal feeding: a review. J Clean Prod. (2014) 65:16–27. 10.1016/j.jclepro.2013.11.068

[70] Narzari S Analysis of Nutritional Value and Biochemical Evaluation of Proteins of Wild Edible Insects Consumed by the Bodos of Selected Areas of Assam. Doctoral dissertation, Department of Biotechnology, Bodoland University, Deborgaon, India. Available online at: http://hdl.handle.net/10603/206529 (accessed September 15, 2020).

[71] Musundire R Zvidzai CJ Chidewe C Samende BK Chemura A Habitats and nutritional composition of selected edible insects in Zimbabwe. JIFF. (2016) 2:189–98. 10.3920/JIFF2015.0083

[72] Bukkens SG The nutritional value of edible insects. Ecol Food Nutr. (1997) 36:287–319. 10.1080/03670244.1997.9991521

[73] Raksakantong P Meeso N Kubola J Siriamornpun S Fatty acids and proximate composition of eight Thai edible terricolous insects. Food Res Int. (2010) 43:350–5. 10.1016/j.foodres.2009.10.014

[74] Mcdonald P Edwards RA Greenhalgh JFD Morgan CA Animal Nutrition, 5th Edn. Harlow: Longman Scientific and Technical (1995).

[75] Yhoung-aree J Viwatpanich K Edible insects in the lao PDR, Myanmar, Thailand and Vietnam. In: Paoletti MG, editors. Ecological Implications of Minilivestock: Potential of Insects, Rodents, Frogs and Snails. Enfield, NH: Science Publishers Inc (2005) 415–40.

[76] Jongjaithet N Wacharangkoon P Paomueng Prapasiri P Protein Quality and Fat Content in Common Edible Insects. Division of Nutrition, Ministry of Public Health. (MOPH). (2008) Available online at: http://nutrition.anamai.moph.go.th/temp/main/view.php?group=3&id=120/ (accessed September 20, 2020).

[77] Joint FAO/WHO Expert Committee on Food Additives Evaluation of Certain Food Additives and Contaminants: Sixty-first report of the Joint FAO/WHO Expert Committee on Food Additives (Report 922). Geneva: World Health Organization (2004).

[78] Payne CLR Scarborough P Rayner M Nonaka K. Are edible insects more or less “healthy” than commonly consumed meats? A comparison using two nutrient profiling models developed to combat over-and undernutrition. Eur J Clin Nutr. (2016) 70:285–91. 10.1038/ejcn.2015.149 26373961 PMC4781901

[79] Finke MD Oonincx D Insects as food for insectivores. In: Morales-Ramos J Rojas G Shapiro-Ilan DI, editors. Mass Production of Beneficial Organisms: Invertebrates and Entomopathogens. New York, NY: Academic Press (2014). p. 583–616. 10.1016/B978-0-12-391453-8.00017-0

[80] Musundire R Zvidzai CJ Chidewe C Samende BK Manditsera FA Nutrient and anti-nutrient composition of *Henicus whellani* (Orthoptera: Stenopelmatidae), an edible ground cricket, in south-eastern Zimbabwe. Int J Trop Insect Sci. (2014) 34:223–31. 10.1017/S1742758414000484

[81] Bednárová M Possibilities of using insects as food in the Czech republic. (Doctoral's Thesis), Mendel University: Brno, Czech Republic (2013).

[82] Poelaert C Francis F Alabi T Megido RC Crahay B Bindelle J Protein value of two insects, subjected to various heat treatments, using growing rats and the protein digestibility-corrected amino acid score. JIFF. (2018) 4:77–87. 10.3920/JIFF2017.0003

[83] Ramos-Elorduy J Moreno JMP Prado EE Perez MA Otero JL De Guevara OL Nutritional value of edible insects from the state of Oaxaca, Mexico. J Food Compos Anal. (1997) 10:142–57. 10.1006/jfca.1997.0530

[84] Finke MD Nutrient content of insects. In: Capinera JL, editor. Encyclopedia of Ento-Mology. Dordrecht: Kluwer Academic (2004). p. 1562–1575.

[85] Bruce RH Allen WK Edwin TM John DA Effect of cooking on the protein profiles and *in vitro* digestibility of sorghum and maize. J Agric Food Chem. (1986) 34:647–9. 10.1021/jf00070a014

[86] Klunder HC Wolkers-Rooijackers J Korpela JM Nout MJR Microbiological aspects of processing and storage of edible insects. Food Control. (2012) 26:628–31. 10.1016/j.foodcont.2012.02.013

[87] Xiaoming C Ying F Hong Z Review of the nutritive value of edible insects. Edible insects and other invertebrates in Australia: future prospects. In: Proceedings of a Workshop on Asia-Pacific Resources and their Potential for Development, 19–21 February 2008. Bangkok (2010). p. 85–92.

[88] Tzompa-Sosa DA Yi L van Valenberg HJ van Boekel MA Lakemond CM Insect lipid profile: aqueous versus organic solvent-based extraction methods. Food Res Int. (2014) 62:1087–94. 10.1016/j.foodres.2014.05.052

[89] Ekpo KE Onigbinde AO Asia IO Pharmaceutical potentials of the oils of some popular insects consumed in southern Nigeria. Afr J Pharm Pharmacol. (2009) 3:51–7. 10.5897/AJPP.9000216

[90] Paoletti MG Norberto L Damini R Musumeci S. Human gastric juice contains chitinase that can degrade chitin. Ann Nutr Metab. (2007) 51:244–51. 10.1159/000104144 17587796

[91] Finke MD. Estimate of chitin in raw whole insects. Zoo Biol. (2007) 26:105–15. 10.1002/zoo.20123 19360565

[92] Muzzarelli RAA Terbojevich M Muzzarelli C Miliani M Francescangeli O (2001). Partial depolymerization of chitosan with the aid of papain. In Muzzarelli RAA editor. Chitin Enzymol. 405–14.

[93] Muzzarelli RA. Chitins and chitosans as immunoadjuvants and non-allergenic drug carriers. Mar Drugs. (2010) 8:292–312. 10.3390/md8020292 20390107 PMC2852840

[94] Lee KP Simpson SJ Wilson K Dietary protein-quality influences melanization and immune function in an insect. Funct Ecol. (2008) 22:1052–61. 10.1111/j.1365-2435.2008.01459.x

[95] Stull VJ Finer E Bergmans RS Febvre HP Longhurst C Manter DK. Impact of edible cricket consumption on gut microbiota in healthy adults, a double-blind, randomized crossover trial. Scient Rep. (2018) 8:10762. 10.1038/s41598-018-29032-2 30018370 PMC6050247

[96] Jarett JK Carlson A Serao MCR Strickland J Serfilippi L Ganz HH. Diets containing edible cricket support a healthy gut microbiome in dogs (No. e27677v1). PeerJ Preprints. (2019) Available online at: https://peerj.com/preprints/27677/ (accessed Saptember 22, 2020). 10.7287/peerj.preprints.27677

[97] Nation JL Insect Physiology and Biochemistry. Boca Raton, Fla: CRC Press (2001) 485p. 10.1201/9781420058376

[98] El-Damanhouri HIH Studies on the influence of different diets and rearing conditions on the development and growth of the two-spotted cricket Gryllus bimaculatus de Geer (Doctoral dissertation). (2011) Available online at: https://epub.uni-bayreuth.de/id/eprint/310 (accessed August 15, 2020).

[99] Maklakov AA Simpson SJ Zajitschek F Hall MD Dessmann J Clissold FJ. Sex-specific fitness effects of nutrient intake on reproduction and lifespan. J Curr Biol. (2008) 18:1062–6. 10.1016/j.cub.2008.06.059 18635354

[100] Harrison SJ Reubenheimer D Simpson SJ Godin J-GJ Bertram SM. Towards a synthesis of framework in nutritional ecology: interacting effects of protein, carbohydrates and phosphorous on field cricket fitness. Proc R Soc B. (2014) 281:20140539. 10.1098/rspb.20140539 25143029 PMC4150310

[101] Anankware JP Osekre EA Obeng-Ofori D Khamala C Identification and classification of common edible insects in Ghana. Int J Entomol Res. (2016) 1:33–9. 10.3920/JIFF2016.0007

[102] Bednárová M Borkovcová M Mlček J Rop O Zeman L Edible insects-species suitable for entomophagy under condition of Czech Republic. Acta Univ Agric Silvic Mendelianae Brun. (2013) 61:587–93. 10.11118/actaun201361030587

[103] FAO/WHO/UNU Energy and protein requirements: report of a Joint FAO/WHO/UNU Expert Consultation [held in Rome from 5 to 17 October 1981]. In Technical Report Series (WHO) World Health Organization (1985) (No. 724), Geneva: World Health Organization (1985). 3937340

[104] Makkar HP Tran G Heuzé V Ankers P State-of-the-art on use of insects as animal feed. Feed Sci Technol. (2014) 197:1–33. 10.1016/j.anifeedsci.2014.07.008

[105] Finke MD. Complete nutrient composition of commercially raised invertebrates used as food for insectivores. Zoo Bio. (2002) 21:269–85. 10.1002/zoo.1003126366856

[106] Cai ZW Zhao XF Jiang XL Yao YC Zhao CJ Xu NY Comparison of muscle amino acid and fatty acid composition of castrated and uncastrated male pigs at different slaughter ages. Ital J Anim Sci. (2010) 9:e33 10.4081/ijas.2010.e33

[107] Ssepuuya G Smets R Nakimbugwe D Van Der Borght M Claes J. Nutrient composition of the long-horned grasshopper *Ruspolia differens* serville: effect of swarming season and sourcing geographical area. Food Chem. (2019) 301:125305. 10.1016/j.foodchem.2019.125305 31387042

[108] Strakova E Suchý PA Vitula FR Večerek VL Differences in the amino acid composition of muscles from pheasant and broiler chickens. Archiv Fur Tierzucht. (2006) 49:508–14. 10.5194/aab-49-508-2006

[109] Gropper SS Smith JL Carr TP Advanced Nutrition and Human Metabolism. 7th ed Boston, MA: Cengage Learning (2016).

[110] van Huis A Oonincx DG The environmental sustainability of insects as food and feed. A review. Agron Sustain Dev. (2017) 37:43 10.1007/s13593-017-0452-8

[111] Bednárová M Borkovcová M Komprda T. Purine derivate content and amino acid profile in larval stages of three edible insects. J Sci Food Agric. (2014) 94:71–6. 10.1002/jsfa.6198 23633284

[112] Žlender B Holcman A Stibilj V Polak T Fatty acid composition of poultry meat from free range rearing. Poljoprivreda Osijek. (2000) 6:53–6.

[113] De Foliart GR The Human Use of Insects as a Food Resource: a Bibliographic Account in Progress. University of Wisconsin. (2002) Available online at: http://food-insects.com/human-use-insects-food-resource-bibliographic-account-progress/ (accessed August 15, 2020).

[114] Bukkens SGF Insects in the human diet: nutritional aspects. In: Paoletti MG, editor. Ecological Implications of Minilivestock: Role of Rodents, Frogs, Snails, and Insects for Sustainable Development. Enfield, CT: Science Publishers Inc (2005). p. 545–77.

[115] Aman P Michel F Megido RC Alabi T Malik P Uyttenbroeck R Insect fatty acids: a comparison of lipids from three orthopterans and *Tenebrio molitor* L. larvae. J Asia-Pac Entomol. (2017) 20:337–40. 10.1016/j.aspen.2017.02.001

[116] Banjo AD Lawal OA Songonuga EA The nutritional value of fourteen species of edible insects in southwestern Nigeria. Afr J Biotechnol. (2006) 5:298–301. 10.5897/AJB05.250

[117] Ajai AI Bankole M Jacob JO Audu UA Determination of some essential minerals in selected edible insects. Afr J Pure Appl Chem. (2013) 7:194–7. 10.5897/AJPAC2013.0504

[118] Hautrive TP Marques A Kubota EH Avaliação da composição centesimal, colesterol e perfil de ácidos graxos de cortes cárneos comerciais de avestruz, suíno, bovino e frango (evaluation of the centesimal composition, cholesterol and fatty acid profile of commercial Ostrich, Swine, Cattle And Chicken Cuts) determination of the composition, cholesterol and fatty acid profi le of cuts of meat trade ostrich. Alimentos e Nutr. Araraquara. (2013) 23:327–34.

[119] Strain JJ Cashman K Minerals and trace elements. In: Gibney MJ Lanham-New SA Cassidy A Vorster HH, editors. Introduction to Human Nutrition. 2nd ed West Sussex: John Wiley & Sons Ltd. (2009). p. 386.

[120] Stipanuk M Caudill M Biochemical, Physiological, and Molecular Aspects of Human Nutrition. 3rd ed St. Louis, MI: Elsevier Health Sciences (2013). p. 753–849.

[121] National Institutes of Health Dietary Supplement Label Database (DSLD). (2017). Available at: https://dsld.od.nih.gov/dsld/ (accessed September 25, 2020).

[122] Christensen DL Orech FO Mungai MN Larsen T Friis H Aagaard-Hansen J. Entomophagy among the Luo of Kenya: a potential mineral source? Int J Food Sci Nutr. (2006) 57:198–203. 10.1080/09637480600738252 17127470

[123] Tomovic V Jokanovic M Sojic B Skaljac S Tasic T Ikonic P Minerals in pork meat and edible offal. Proc Food Sci. (2015) 5:293–5. 10.1016/j.profoo.2015.09.083

[124] National Institute of Industrial Research, NIIR The Complete Technology Book on Meat, Poultry and Fish Processing. New Delhi: NIIR Project Consultancy Services (2008). p. 1–488.

[125] Hurrell R Egli I. Iron bioavailability and dietary reference values. Am J Clin Nutr. (2010) 91:1461S−7S. 10.3945/ajcn.2010.28674F 20200263

[126] de Castro RJS Ohara A dos Santos Aguilar JG Domingues MAF Nutritional, functional and biological properties of insect proteins: processes for obtaining, consumption and future challenges. Trends Food Sci Technol. (2018) 76:82–9. 10.1016/j.tifs.2018.04.006

[127] Gladys Latunde-Dada O Yang W Vera Aviles M. In vitro iron availability from insects and sirloin beef. J Agr Food Chem. (2016) 64, 8420–4. 10.1021/acs.jafc.6b03286 27731991

[128] Institute of Medicine. Standing Committee on the Scientific Evaluation of Dietary Reference Intakes and its Panel on Folate, Other B Vitamins, and Choline. Dietary Reference Intakes for Thiamin, Riboflavin, Niacin, Vitamin B6, Folate, Vitamin B12, Pantothenic Acid, Biotin, and Choline. Washington (DC): National Academies Press (US) (1998). 23193625

[129] Borkovcová M Bednárová M Fišer V Ocknecht P Kitchen Variegated by Insects 1. Lynx, Brno (2009).

[130] Ramos-Elorduy J Menzel P Creepy Crawly Cuisine: the Gourmet Guide to Edible Insects. Inner Traditions/Bear and Co. Park Street Press: South Paris (1998).

[131] Rosner F. Pigeons as a remedy (segulah) for jaundice. N Y State J Med. (1992) 92:189–92. 1614669

[132] Souza-Dias JP Índice de drogas medicinais angolanas em documentos dos séculos XVI a XVIII. Rev Port Farm. (1995) 45:174–84.

[133] Unnikrishnan PM Animals in ayurveda. Amruth. (1998) 1:1–23.

[134] Rajkhowa D Rokozeno DMK Insect-based medicine: a review of present status and prospects of entomo-therapeutic. IJAEB. (2016) 9:1069–79. 10.5958/2230-732X.2016.00135.2

[135] Ahn MY Han JW Hwang JS Yun EY Lee BM. Anti-inflammatory effect of glycosaminoglycan derived from *Gryllus bimaculatus* (a type of cricket, insect) on adjuvant-treated chronic arthritis rat model. J Toxicol Environ A. (2014) 77:1332–45. 10.1080/15287394.2014.951591 25343284

[136] De Asis FYTF Historia de la Medicina en México desde la época de los indios hasta el presente. México. Ed. Facsimilar Secretaria de Fomento IMSS IV Vols. 2819. Madrid: México, Oficina tip de la Secretaría de fomento (1982) 1886–88.

[137] Barajas CLE Los animales usadosen la medicina popular mexicana. (Thesis Prof). Fac. de Gencias, UNAM (1951).

[138] de Conconl JRE Los insectos como una fuente de proteinas en el futuro. Limusa Mexico (1982). p. 142.

[139] Fosaranti JO The place of insects in the traditional medicine of southwestern Nigeria. Food Insects Newsletter. (1997) 10:1–5.

[140] Banjo AD Lawal OA Fapojuwo Songonuga OEEA Farmers' knowledge and perception of horticultural insect pest problems in southwestern Nigeria. Afr J Biotechnol. (2003) 2:434–7. Available online at: http://www.academicjournals.org/AJB

[141] Bozhou Sawnf Commerce and Trade CO LTD Natural Dried Wild Gryllolaptaptidae Chinese Mole Cricket Insects for Food. (2020) Available online at: https://www.alibaba.com/product-detail/Natural-dried-wild-Gryllolaptaptidae-chinese-mole_60817265136.html (accessed September 1, 2020).

[142] Kipkoech C Kinyuru JN Imathiu S Roos N Use of house cricket to address food security in Kenya: nutrient and chitin composition of farmed crickets as influenced by age. Afr J Agric Res. (2017) 12:3189–97. 10.5897/AJAR2017.12687

[143] Ahn MY Lee YW Ryu KS Lee HS Kim IS Kim JW Effects of water and methanol extracts of cricket (*Gryllus bimaculatus*) on alcohol metabolism. Kor J Pharmacogn. (2004) 35:175–8.

[144] Hwang BB Chang MH Lee JH Heo W Kim JK Pan JH. The edible insect *Gryllus bimaculatus* protects against gut-derived inflammatory responses and liver damage in mice after acute alcohol exposure. Nutrients. (2019) 11:857. 10.3390/nu11040857 30995745 PMC6521266

[145] Ahn MY Hwang JS Yun EY Gene expression profiling of glycosaminoglycan drived from *G. bimaculatus* in high fat dieted rat. FASEB J. (2015) 29(1Suppl.):LB152.

[146] Halloran A Hanboonsong Y Roos N Bruun S Life cycle assessment of cricket farming in north-eastern Thailand. J Clean Prod. (2017) 156:83–94. 10.1016/j.jclepro.2017.04.017

[147] Van Huis A Van Itterbeeck J Klunder H Mertens E Halloran A Vantomme P Edible insects: Future prospects for food and feed security. Rome: Food and Agriculture Organization of the United Nations (2013). p. 187. Available online at: http://www.fao.org/docrep/018/i3253e/i3253e14.pdf

[148] Raheem D Carrascosa C Oluwole OB Nieuwland M Saraiva A Millán R. Traditional consumption of and rearing edible insects in Africa, Asia and Europe. Crit Rev Food Sci Nutr. (2018) 59:2169–88. 10.1080/10408398.2018.1440191 29446643

[149] Fuah AM Siregar HCH Endrawati YC Cricket farming for animal protein as profitable business for small farmers in Indonesia. J Agric Sci Technol. (2015) 5:296–304. 10.17265/2161-6256/2015.04.008

[150] Abdul Razak I Yusof HA Engku AEA Nutritional evaluation of house cricket (*Brachytrupes portentosus*) meal for poultry. In: 7th proceedings of the Seminar in Veterinary Sciences;27 February–March 2. Selangor (2012). p. 14–18.

[151] Taufek NM Simarani K Muin H Aspani F Raji AA Alias Z. Inclusion of cricket (*Gryllus bimaculatus*) meal in African catfish (*Clarias gariepinus*) feed influences disease resistance. J Fisheries. (2018) 6:623–31. 10.17017/jfish.v6i2.2018.26426886132

[152] Taufek NM Muin H Raji AA Md Yusof H Alias Z Razak SA. Potential of field crickets meal (*Gryllus bimaculatus*) in the diet of African catfish (*Clarias gariepinus*). J Appl Anim Res. (2018) 46:541–6. 10.1080/09712119.2017.135756026886132

[153] Wang D Zhai SW Zhang CX Bai YY An SH Xu YN Evaluation on nutritional value of field crickets as a poultry feedstuff. Asian-Australas J Anim Sci. (2005) 18:667–70. 10.5713/ajas.2005.667

[154] Schiavone A Cullere M De Marco M Meneguz M Biasato I Bergagna S. Partial or total replacement of soybean oil by black soldier fly larvae (Hermetia illucens L.) fat in broiler diets: effect on growth performances, feed choice, blood traits, carcass characteristics and meat quality. Ital J Anim Sci. (2017) 6:93–100. 10.1080/1828051X.2016.124996826220968

[155] Cerritos R Cano-Santana Z Harvesting grasshoppers *Sphenarium purpurascens* in Mexico for human consumption: a comparison with insecticidal control for managing pest outbreaks. Crop. Prot. (2008) 27:473–80. 10.1016/j.cropro.2007.08.001

[156] Cerritos R Insects as food: an ecological, social and economical approach. CAB Rev. (2009) 4:1–10. 10.1079/PAVSNNR20094027

[157] Hoare AL The Use of Non-Timber Forest Products in the Congo Basin: Constraints and Opportunities. New York, NY: Rainforest Foundation. (2007) Available online at: http://tinyurl.com/oyqohag (accessed August 20, 2020).

[158] Adriaens EL Recherches sur l'alimentation des populations au Kwango. Bull Agric Congo Belge. (1951) 62:473–550.

[159] Hanboonsong Y Durst PB Edible Insects in Lao PDR: Building on Tradition to Enhance Food Security. Food and Agriculture Organization of the United Nations: Bangkok, Thailand (2014). p. 55.

[160] Osimani A Milanović V Cardinali F Roncolini A Garofalo C Clementi F Bread enriched with cricket powder (*Acheta domesticus*): a technological, microbiological and nutritional evaluation. Innov Food Sci Emerg Technol. (2018) 48:150–63. 10.1016/j.ifset.2018.06.007

[161] FAO Edible Insects—Future Prospects for Food and Feed Security. FAO Forestry Paper, Vol 171. Rome: Food and Agriculture Organization of the United Nations (2013).

[162] Van der Meer Mohr JVD Insects eaten by the Karo-Batak people (a contribution to entomo-bromatology). Entomol Berichten Amster. (1965) 25:101–7.

[163] Zielińska E Karaś M Jakubczyk A Antioxidant activity of predigested protein obtained from a range of farmed edible insects *IJST*. (2017) 52:306–12. 10.1111/ijfs.13282

[164] Portes E Gardrat C Castellan A Coma V Environmentally friendly films based on chitosan and tetrahydrocurcuminoid derivatives exhibiting antibacterial and antioxidative properties. Carbohyd Polym. (2009) 76:578–84. 10.1016/j.carbpol.2008.11.031

[165] Cutter CN. Opportunities for bio-based packaging technologies to improve the quality and safety of fresh and further processed muscle foods. Meat Sci. (2006) 74:131–42. 10.1016/j.meatsci.2006.04.02 22062722

[166] Weidner H Insekten im Volkskunde und Kulturgeschichte. Arbeitsgemeinschaft der Museen in Schleswig-Holstein. Niederschrift uber die Tagung der Arbeitsgemeinschaft am 28. und 29. Oktober 1950 im Heimatsmuseum in Rendsburg. (1952). p. 33–45.

[167] Ryan LG Insect Musicians and Cricket Champions: a Cultural History of Singing Insects in China and Japan. San Francisco, CA: China Books and Periodicals, Inc. (1996).

[168] Laufer B Insect-musicians and cricket champions of China. Field Mus Nat Hist. (1927) 22:1–27.

[169] Bidau CJ The katydid that was: the tananá, stridulation, Henry Walter Bates and Charles Darwin. Arch Nat Hist. (2014) 41:131–40. 10.3366/anh.2014.0216

[170] Jacobs A Chirps and Cheers: China's Crickets Clash. (2011) Available online at: https://www.nytimes.com/2011/11/06/world/asia/chirps-and-cheers-chinas-crickets-clash-and-bets-are-made.html (accessed September 22, 2020).

[171] Xing-Bao J Kai-Ling X An index-catalogue of Chinese tettigoniodea (Orthopteroidea: Grylloptera). J Orthoptera Res. (1994) 163:15–41. 10.2307/3503405

[172] Hogue CL Cultural entomology. Ann Rev Ent. (1987) 32:181–99.

[173] Kritsky G Cherry RH Insect Mythology. iUniverse. Lincoln: Writers Club Press (2000). p. 140.

[174] Carrera M Insetos, lendas e história. Brasília: DF (1991). p. 137.

[175] Forde GA Folk Beliefs of Barbados. Barbados: The National Cultural Foundation (1988). p. 47.

[176] Lenko K Papavero N Insetos no Folclore. São Paulo, Brazil: Plêiade/FAPESP (1996). p. 468.

[177] Araújo AM Medicina Rústica. São Paulo: Companhia Editora Nacional (1959). p. 301.

[178] Costa-Neto EM. “Cricket singing means rain”: semiotic meaning of insects in the district of Pedra Branca, Bahia State, northeastern Brazil. An Acad Bras Cienc. (2006) 78:59–68. 10.1590/S0001-37652006000100007 16532207

[179] Fowler H Canibalismo entre insetos. Ciência Hoje. (1994) 18:15–6.

[180] Mbata KJ Traditional uses of arthropods in Zambia: II. Medicinal and miscellaneous uses. Food Insects Newsl. (1999) 12:1–7.

[181] Bray A. Vietnam's Most Challenging Foods -To Much of the World They're Pests to be Exterminated or Animal Parts to be Thrown Out; in Vietnam They all go Into the Cooking Pot. (2010) Available online at: http://travel.cnn.com/explorations/eat/vietnams-bizarre-foods-864722/ (accessed August 15, 2020).

[182] Bednárová M Borkovcová M Zorníková G Zeman L Insect as food in Czech republic. In: Proceedings Mendel Net, 24 November 2010. Brno: Mendel University (2010). p. 674–82.

[183] Zagrobelny M Dreon AL Gomiero T Marcazzan GL Glaring MA Møller BL Toxic moths: source of a truly safe delicacy. J Ethnobiol. (2009) 29:64–76. 10.2993/0278-0771-29.1.64

[184] Islamiyat FB Moruf OO Sulaiman AO Adeladun SA A review of cyanogenic glycosides in edible plants. In: Larramendy M Soloneski S, editors. Toxicology-New Aspects to This Scientific Conundrum. (2016). p. 180–186. Available online at: https://www.intechopen.com/books/toxicology-new-aspects-to-this-scientific-conundrum/a-review-of-cyanogenic-glycosides-in-edible-plants (accessed September 10, 2020).

[185] Bouvier G. Quelques questions d'entomologie vétérinaire et lutte contre certains arthropodes en Afrique tropicale [Some questions of veterinary entomology and the fight against certain arthropods in tropical Africa]. Acta Trop. (1945) 2:42–59. 21008127

[186] Reineke K Doehner I Schlumbach K Baier D Mathys A Knorr D The different pathways of spore germination and inactivation in dependence of pressure and temperature. Food Sci Emerg Technol. (2012) 13:31–41. 10.1016/j.ifset.2011.09.006

[187] Giaccone V Hygiene and health features of minilivestock. In: Paoletti MG, editor. Ecological Implications of Minilivestock: Potential of Insects, Rodents, Frogs and Snails. Enfield NH: Science Publisher (2005). p. 579–98.

[188] Hanboonsong Y Edible Insect Recipes: Edible Insects for Better Nutrition and Improved Food Security. Vientiane: FAO and the Government of Lao PDR (2012).

[189] Megido RC Alabi T Nieus C Blecker C Danthine S Bogaert J Optimisation of a cheap and residential small-scale production of edible crickets with local by-products as an alternative protein-rich human food source in ratanakiri province, Cambodia. J Sci Food Agric. (2016) 96:627–32. 10.1002/jsfa.7133 25683556

[190] Hanboonsong Y Rattanapan A Utsunomiya Y Masumoto K Edible insects and insect-eating habits in northeastern Thailand. Elytra. (2000) 28:355–64.

[191] Schmitschek E Insekten als nahrung, in brauchtum, kult und kultur. handbuch der zoologie-eine naturgeschichte der stämme des tierreichs. In: Helmcke JG Stark D Wermuth H, editors. Handbuch der Zoologie- eine Naturgeschichte der Stamme des Tierreichs, Band 4. Akademie Verlag: Berlin Bd (1968). p. 1–62.

[192] Kvassay G The complete cricket breeding manual: revolutionary new cricket breeding systems. Zega Enterprises New South Wales. 1812.

[193] Reverberi M Edible insects: cricket farming and processing as an emerging market. JIFF. (2020) 6:211–20. 10.3920/JIFF2019.0052

[194] Hanboonsong Y Edible insects and associated food habits in Thailand. Forest Insects Food Humans Bite Back. (2010) 2:173–182.

[195] Meyer-Rochow VB Ghosh S Jung C Farming of Insects for Food and Feed in South Korea: Tradition and Innovation. Berliner und Münchener Tierärztliche Wochenschrift (2018). 10.2376/0005-9366-18056. Available online at: https://www.vetline.de/farming-of-insects-for-food-and-feed-in-south-korea-tradition-and-innovation

[196] VandalSoft A New Innovation in the Edible Insect Smart Farm. (2018) Available online at: http://vandalsoft.com/ (accessed September 1, 2020).

[197] Chung AYC Khen CV Unchi S Binti M Edible insects and entomophagy in Sabah, Malaysia. Malay Nat J. (2002)56:131–44.

[198] Adalla CB Cervancia CR Philippine edible insects: a new opportunity to bridge the protein gap of resource-poor families and to manage pests. In: Durst PB Johnson DV Leslie RN Shono K, editors. Forest Insects as Food: Humans Bite Back. Proceedings of a Workshop on Asia-Pacific Resources and Their Potential for Development, Chiang Mai, Thailand, 19–21 February, 2008. Bangkok, Thailand: FAO (2010). p. 151–160.

[199] EntomoFarms The Planet's Most Sustainable Super-Food. (2020) Available online at: https://entomofarms.com/home/ (accessed September 1, 2020).

[200] Castaldo J Change Agents 2016: Isha Datar, New Harvest: Reinventing the Way Meat is Made. (2016) Available online at: https://www.canadianbusiness.com/innovation/change-agent/isha-datar-new-harvest/ (accessed September 1, 2020).

[201] Van Itterbeeck J Rakotomalala Andrianavalona IN Rajemison FI Rakotondrasoa JF Ralantoarinaivo VR Hugel S. Diversity and use of edible grasshoppers, locusts, crickets, and katydids (Orthoptera) in Madagascar. Foods. (2019) 8:666. 10.3390/foods8120666 31835637 PMC6963331

[202] Stein C Florence D Yacouba K Kariba C Stefan J Potential approach to regulate and monitor moisture for Brachytrupes membreneus eggs for cricket rearing in the village of sanambele. Mali Poster. 1.

[203] Magara JOH Tanga CM Ayieko MA Hugel S Coopeland RS Samira AM Effect of rearing substrates on the fitness parameters of newly recorded edible cricket *Scapsipedus marginatus* in Kenya. JIFF. (2018) 4. 10.3920/JIFF2018.S1

[204] Otieno MH Ayieko MA Niassy S Salifu D Abdelmutalab AG Fathiya KM. Integrating temperature-dependent life table data into insect life cycle model for predicting the potential distribution of *Scapsipedus icipe* Hugel and Tanga. PLoS ONE. (2019) 14:e0222941. 10.1371/journal.pone.0222941 31553778 PMC6760797

[205] Smart Harvest by Reuters Cricket-Farming Hops Ahead as Kenyans Catch Superfood Bug. (2018) Available online at: https://www.standardmedia.co.ke/farmkenya/article/2001297031/cricket-farming-hops-ahead-as-kenyans-catch-superfood%20bug (accessed September 1, 2020).

