# Supplementary material for: Edible Crickets (Orthoptera) Around the World: Distribution, Nutritional Value, and Other Benefits—A Review
Source: Front Nutr. 2021 Jan 12;7:537915. doi: 10.3389/fnut.2020.537915 (PMC7835793; doi:10.3389/fnut.2020.537915)
Supplement: Supplementary file 1 [file Table_1.pdf]

Table 1 Common edible cricket species in the world

Cricket						
**Taxonomic position**	**Common name**	**Latin name**	**Stage consumed**	**Distribution**	**Source of**	**Literature source**
**Gryllotalpidae**	Mole cricket	*Gryllotalpa africana* Palisot de Beauvois	Adult /nymph	**Asia** Thailand, China, Philippines, Vietnam, India, Indonesia, Laos People's Democratic Republic<br><br>**Africa** Uganda, Zimbabwe	Food	Hanboonsong, 2000; Lumsa-ad 2001; Weiping et al., 2017; Dele et al., 2018; Bray, 2010 ; Yhoung-Aree et al., 2005; Van der Meer Mohr, 1965; Hanboonsong and Durst, 2014; DeFoliart, 2002; Jongema, 2017<br><br>Bodenheimer,1951; Chavanduka, 1975; Gelfand, 1971; Weaving, 1973
**Gryllotalpidae**	African mole cricket	*Gryllotalpa africana microphtalma* Chopard	adult	**Asia** Thailand,<br><br>**Africa** Nigeria	Food	Jongema, 2017; Banjo et al., 2006
**Gryllotalpidae**	Mole cricket	*Gryllotalpa orientalis* Burmeister (Figure 5)	Adult	**Asia** China, Vietnam	Food	Weiping et al., 2017; Dele et al., 2018; Bray, A. 2010; Chen and Feng, 2009
**Gryllotalpidae**	Mole cricket	*Gryllotalpa unispina* Saussure	Adult	**Asia** China, Vietnam, Japan	Food	Weiping et al., 2017; Dele et al., 2018; Bray, 2010; Schimitschek, 1968

**Gryllotalpidae**	Mole cricket	*Gryllotalpa hirsuta* Burmeister	Adult	**Asia** Malaysia, Sabah	Food	Chung et al., 2002
**Gryllotalpidae**	Mole cricket	*Gryllotalpa longipennis* de Haan	adult	**Asia** Malaysia, Sabah	Food	Chung et al., 2002
**Gryllotalpidae**	Mole cricket	*Gryllotalpa* sp.	Adult	**Asia** Indonesia, Philippines, India	Food	DeFoliart, 2002; Adalla et al., 2010; Singh et al., 2007; Chakravorty et al., 2011
**Gryllotalpidae**	Mole cricket	*Gryllotalpa* sp.	Nymph/ Adult	**Australia** Papua New Guinea	Food	DeFoliart, 2002; Jongema, 2017
**Gryllotalpidae**	Mole cricket	*Neoscapteriscus vicinus* (Scudder) under *Scapteriscus vicinus* Scudder	Adult	**Asia** Vietnam<br>**Africa** Ghana	food	Anankware et al., 2016; Dele et al., 2018; Bray, 2010
**Gryllotalpidae**	Mole cricket	*Gryllotalpidae* gen.	Adult	**America** Mexico	Food	DeFoliart, 2002; Jongema, 2017
**Trigonidiidae**		*Pteronemobius* sp.	Adult	**Asia** Thailand	Food	Hanboonsong, 2010; Jongema, 2017
**Trigonidiidae**	Striped ground cricket	*Allonemobius fasciatus* (De Geer)	Adult	**Asia** Vietnam	Food	Dele et al., 2018; Bray, A. 2010
**Trigonidiidae**	Christmas cricket	*Homoeoxipha* sp.	Adult	**Asia** Thailand	Food	Hanboonsong, 2010; Jongema, 2017

**Gryllidae**	House cricket	*Acheta domesticus* Linnaeus (Figure 6)	Adult	**Asia**<br>Thailand, china, Cambodia , Lao People's Democratic Republic<br><br>**Africa**<br>Kenya, Ghana Cameroon, The Central African Republic, Democratic Republic of Congo Zambia, Zimbabwe<br><br>**Europe**<br>Netherland Belgium, Switzerland<br><br>**America**<br>Mexico<br>USA,<br>**Australia**<br>Papua New Guinea, New Zealand	Food/ feed	Yhoung-Aree and Viwat panich, 2005; van Huis et al., 2013; Orinda, 2018; EFSA, 2015; Anankware et al., 2016; Megido et al. 2016; Dele et al., 2018; Kelemu et al., 2015; Chavanduka , 1976; Mbata, 1995; Halloran et al., 2017; Jongema, 2017; Angie; 2019; Instar Farming. 2020; Ramos- Elorduy, 2009
**Gryllidae**	House cricket	*Gryllus testaceus* Walker	Adult	**Asia**<br>Thailand,	Food/ Feed	Jongjaithet et al., (2008); Yhoung-aree and

				Lao People's Democratic Republic, Myanmar (Burma) Vietnam, Malaysia		Viwatpanich (2005; Taufek et al., 2018
**Gryllidae**	Ground cricket	*Modicogryllus* (*Modicogryllus*) *confirmatus* *Acheta confirmata*	Adult	**Asia** Thailand	Food	Dele et al., 2018, Tang et al. 2019
**Gryllidae**	Spring cricket	*Gryllus veletis* (Alexander and Bigelow)	Adult	**America** North America	Feed	van Huis et al., 2013
**Gryllidae**	Field cricket	*Eumodicogryllus bordigalensis* (Latreille) under *Gryllus chinensis* Weber	Adult	**Asia** China	Food	Weiping et al., 2017
**Gryllidae**	Two-spotted black cricket	*Gryllus bimaculatus* DeGeer (Figure 4)	Adult	**Asia** Thailand, South Korea, Indonesia, Malaysia **Africa** Kenya, Zambia, Guinea Bissau, Sierre Leone, Guinée, Liberia, Benin, Togo, Nigeria,	Food/ feed	van Huis et al., 2013; Orinda, 2018 ; Kelemu et al., 2015; Ayieko et al., 2016; Hwang et al., 2019; Halloran et al., 2017; Mbata, 1995, 2002; Angie, 2019 ; Taufek et al., 2018

				Democratic Republic of Congo, Kenya, South Sudan<br><br>**Australian**<br>New Zealand		
	Jamaican field cricket	*Gryllus assimilis* (Fabricius)	Adult	**Asia**<br>India<br><br>**Africa**<br>North Nigeria<br><br>**Europe**<br>Poland<br><br>**America**<br>North America, Mexico, Brazil,	Food/ Feed	EFSA, 2015; DeFoliart, 2002; Jongema, 2017; Ramos- Elorduy 2009; Araújo et al., 2019; Mlček et al., 2018; Oibiokpa et al., 2017
**Gryllidae**	Field cricket	*Gryllus similis* Chapman (Figure 3)	Adult	**Africa**<br>Ghana	Food	Anankware et al., 2016
**Gryllidae**	Field cricket	*Gryllus* sp.	Adult	**Africa**<br>Benin	Food	Tchibozo, 2005; Jongema, 2017
**Gryllidae**	Banded cricket	*Gryllodes sigillatus* (Walker)	Nymph/ Adult	**Europe**<br>Belgium	Food/ feed	Tang et al., 2019; Józefiak et al., 2016; Weiping et al., 2017; Megido et al., 2016 ; EFSA, 2015
**Gryllidae**	Field cricket	*Gryllodes* sp.	Adult	**Africa**<br>Central	Food	Hoare, 2007; Jongema, 2017

				Africa Republic		
**Gryllidae**	Chinise fighting cricket	*Teleogryllus mitratus* (Burmeister) under *Gryllus testaceus* Walker	Adult	**Asia** China, Cambodia, Thailand,	Food/ feed	Weiping et al., 2017; Wang et al., 2005; Miech et al., 2016; DeFoliart, 2002
**Gryllidae**	Field cricket	*Teleogryllus mitratus* Burmeister	Adult	Asia Thailand, China, Lao People's Democratic Republic, Indonesia	Food	van Huis et al., 2013; Van der Meer Mohr, 1965; Jongema, 2017; Hanboonsong, 2010; Yhoung-Aree et al., 2005
**Gryllidae**	Field cricket	*Teleogryllus mitratus* (Burmeister) under *Gryllus mitratus* Burmeister	Nymph /Adult	**Asia** Japan	Food	Schimitschek, 1968; Jongema, 2017
**Gryllidae**	Field cricket	*Teleogryllus occipitalis* (Serville)	Adult	**Asia** Thailand, China	Food	van Huis et al., 2013; Hanboonsong et al., 2013
**Gryllidae**	Field cricket	*Teleogryllus emma* (Ohmachi and Matsumura)	Adult	Asia South Korea	Feed	van Huis et al., 2013; Gosh et al., 2017
**Gryllidae**	Field cricket	*Teleogryllus derelictus* Gorochov	Nymph/ Adult	**Asia** China	Food	Weiping et al., 2017
**Gryllidae**	Black field cricket	*Teleogryllus commodus* (Walker)	Adult	**Australia** Papua New Guinea	Food	DeFoliart, 2002; Jongema, 2017
**Gryllidae**		*Teleogryllus* sp.		**Asia** Thailand	Food	Hanboonsong, 2010; Jongema, 2017
**Gryllidae**	House cricket	*Scapsipedus icipe* Tanga and Hugel	Adult	**Africa** Kenya	Food	Magara et al., 2019, Otieno et al., 2019; Tanga et al.,

						2018
**Gryllidae**	House cricket	*Scapsipedus marginatus* (Afzelius and Brannius)	Adult	**Africa** Kenya	Food	Magara et al., 2018
**Gryllidae**	Ground cricket	*Gymnogryllus leucostictus* (Burmeister)	adult	**Asia** Indonesia	Food	Van der Meer Mohr, 1965; Jongema, 2017
**Gryllidae**		*Gymnogryllus leucostictus* (Burmeister) under *Gymnogryllus elegans* Guérin-Méneville	adult	**Asia** Indonesia	Food	Van der Meer Mohr, 1965; Jongema, 2017
**Gryllidae**	Cliring cricket	*Gryllus mitratus*	Nymph/ Adult	**Asia** Indonesia	Food/ feed	Fuah et al., 2015
**Gryllidae**		*Gymnogryllus* sp.	adult	**Asia** Thailand	Food	Hanboonsong, 2010; Jongema, 2017
**Gryllidae**		*Phonarellus (Semaphorellus) lucens* (Walker) under *Gymnogryllus lucens* Walker	Adult	**Africa** Niger	Food	Ajai et al., 2013
**Gryllidae**		*Velarifictorus aspersus* (Walker) under *Gryllodes berthellus* Saussure	Adult	**Asia** Japan	Food	Schimitschek, 1968; Jongema, 2017
**Gryllidae**	Burrowing cricket	*Velarifictorus aspersus* (Walker)	Adult	**Asia** Japan	Food	Schimitschek, 1968; Jongema, 2017

**Gryllidae**	Burrowing cricket	*Velarifictorus* sp.	Adult	**Asia** Thailand	Food	Hanboonsong, 2010; Jongema, 2015
**Gryllidae**		*Loxoblemmus arietulus* Saussure	Adult	**Asia** Japan	Food	Schimitschek, 1968; Jongema, 2017
**Gryllidae**		*Loxoblemmus doenitzi* Stein	Adult	**Asia** Japan	Food	Schimitschek, 1968; Jongema, 2017
**Gryllidae**	Field cricket	*Grylloderes melanocephalus* (Serville)	Adult	**Asia** India	Food	DeFoliart, 2002; Jongema, 2017
**Gryllidae**	Giant sand cricket	*Brachytrupes membranaceus* (Drury) (Figure 7)	Nymphs and adults	**Africa** Cameroon, Burkina Faso, Angora, Togo, Kenya Coast Zimbabwe, Benin, Democratic Republic of Congo, Central Africa Republic, Nigeria, Tanzania, East, Central and Southern Africa, Zambia, Mali	Food	Kelemu et al., 2015; Chavanduka, 1976; Gelfand, 1971; Weaving, 1973; Tchibozo et al., 2005; Riggi et al., 2013; Bani, 1995, Nkouka, 1987; Hoare, 2007; Adriaens, 1951, Fasoranti and Ajiboye, 1993; Bodenheimer, 1951; Harris, 1940; Mbata, 1995; Jongema, 2017; Stein et al., 2013
**Gryllidae**		*Brachytrupes* sp.	Adult	**Africa** Uganda	Food	Akullo et al., 2018
**Gryllidae**	Giant cricket	*Tarbinskiellus portentosus* (Lichtenstein)	Nymph/ Adult	**Asia** Thailand, China	Food	Weiping et al., 2017; Hanboonsong, 2010;

						Anonymous, 2019
**Gryllidae**	Short tailed cricket	*Tarbinskiellus portentosus* (Lichtenstein) under *Brachytrupes portentosus* Lichtenstein	Adult	**Asia** Malaysia, Thailand, Lao People's Democratic Republic	Food	Weiping et al., 2017; Y. Hanboonsong, personal communication, 2012; Hanboonsong and Durst 2014; Jongema, 2017; Raksakantong et al., 2010
**Gryllidae**	Giant cricket	*Tarbinskiellus orientalis* (Fabricius)	Adult/ nymph	**Asia** India	Food	Chakravorty et al., 2011; Jongema, 2017
**Gryllidae**	Giant cricket	*Tarbinskiellus* sp.	Adult/ nymph	**Asia** India	Food	Singh et al., 2007; Jongema, 2017
**Gryllidae**	Short tailed cricket	*Anurogryllus arboreus* Walker	Adult	**Asia** Vietnam	Food	Dele et al., 2018; Bray, A. 2010
**Gryllidae**		*Nisitrus vittatus* (deHaan)	Adult	**Asia** Indonesia	Food	Van der Meer Mohr, 1965; Jongema, 2017
**Gryllidae**	Field crickt	*Gryllidae* gen.	Adult	**America** Mexico	Food	DeFoliart, 2002; Jongema, 2017
**Gryllidae**	Burrowing cricket	*Modicogryllus confirmatus* (Walker)	Adult	**Asia** Thailand, Lao People's Democratic Republic	Food	Hanboonsong, 2000; Yhoung-Aree et al., 2005

**Phalangopsidae**		*Amphiacusta annulipes* (Serville)	adult	**Europe** Poland	Food	Zielińska et al., 2017
**Gryllidae**	Ground cricket	*Pteronemobius malagachirus*	adults	**Africa Madagascar**	Food	Van Itterbeeck et al., 2019
**Gryllidae**	Large ground cricket	*Gryllus* sp.	Nymph	**Africa** Madagascar	Food	Van Itterbeeck et al., 2019
**Gryllidae**	Jerman cricket	*Gryllus* sp.	Adult	**Asia** Indonesia	Food	Fuah et al., 2016
**Gryllidae**	Tree cricket	*Fryerius* sp.	Adults	**Africa** Madagascar	Food	Van Itterbeeck et al., 2019
**Gryllidae**	Large ground cricket	*Brachytrupes membranaceus* colosseus (Saussure, 1899)	Adults	**Africa** Madagascar	Food	Van Itterbeeck et al., 2019
**Gryllidae**		*Gryllus campestris*	Adults	**Africa** Burkina Faso	Food	Séré et al., 2018
**Gryllidae**	Field cricket	*Modicogryllus conspersus* (Schaum).	Adults	**Africa** Uganda	Feed and food	Ssepuuya et al., 2020

References

Adalla, C. B., and Cervancia, C. R. (2010). Philippine edible insects: A new opportunity to bridge the protein gap of resource-poor families and to manage pests. In P. B. Durst, D. V. Johnson, R. N. Leslie, & K. Shono (Eds.), Forest insects as food: humans bite back. Proceedings of a workshop on Asia-Pacific resources and their potential for development, Chiang Mai, Thailand, 19–21 February, 2008 (pp. 151–160). Bangkok, Thailand: FAO.

Adriaens E. L. (1951) Recherches sur l'alimentation des populations au Kwango. Bull. agric. Congo belge 62: 473–550.

Ajai, A. I., Bankole, M., Jacob, J. O., Audu, U. A. (2013). Determination of some essential minerals in selected edible insects. Afr. J. Pure Appl. Chem. 7(5):194-197. doi : 10.5897/AJPAC2013.0504

Akullo, J., Agea, J. G., Obaa, B. B., Okwee-Acai, J., Nakimbugwe, D. (2018). Nutrient composition of commonly consumed edible insects in the Lango sub-region of northern Uganda. Int Food Res J. 25(1): 159-166.

Anankware, J. P., Osekre, E. A., Obeng-Ofori, D., Khamala, C. (2016). Identification and classification of common edible insects in Ghana. Int. J. Entomol. Res. 1(5): 33-39.

Angie, S. (2019). Survey reveals our appetite for eating insects. https://www.newshub.co.nz/home/rural/2019/07/survey-reveals-our-appetite-for-eating-insects.html [Accessed August 15, 2020].

Araújo, R. R. S., dos Santos Benfica, T. A. R., Ferraz, V. P., Santos, E. M. (2019). Nutritional composition of insects *Gryllus assimilis* and *Zophobas morio*: Potential foods harvested in Brazil. J. Food Compos. Anal. 76: 22-26. doi: 10.1016/j.jfca.2018.11.005

Ayieko, M. A., Ogola, H. J., Ayieko, I. A. (2016). Introducing rearing crickets (gryllids) at household levels: adoption, processing and nutritional values. JIFF. 2(3): 203-211. doi: 10.3920/JIFF2015.0080

Bani, G. (1995). Some aspects of entomophagy in the Congo. Food Insects Newsl. 8(3): 4-5.

Banjo, A. D., Lawal, O. A., Songonuga, E. A. (2006). The nutritional value of fourteen species of edible insects in southwestern Nigeria. Afr. J. Biotechnol. 5(3): 298-301. doi: 10.5897/AJB05.250

Bodenheimer, F. S. (1951). Insects as human food. In Insects as Human Food (pp. 7-38). Springer, Dordrecht.

Bray, A., (2010). Vietnam's most challenging foods -To much of the world they're pests to be exterminated or animal parts to be thrown out; in Vietnam they all go into the cooking pot. http://travel.cnn.com/explorations/eat/vietnams-bizarre-foods-864722/ [Accessed August 15, 2020].

Chakravorty, J., Ghosh, S., Meyer-Rochow, V. B. (2011). Practices of entomophagy and entomotherapy by members of the Nyishi and Galo tribes, two ethnic groups of the state of Arunachal Pradesh (North-East India). J. Ethnobiol. Ethnomed. 7(1); 5. doi:10.1186/1746-4269-7-5

Chavunduka, D. M. (1975). Insects as a source of protein to the African. Rhodesia Science News, 9: 217-220.

Chen X, Feng Y and Chen Z.-Y. (2009) Common edible insects and their utilization in China. *Entomological Research* 39(5): 299-303.

Chung, A. Y. C., Khen, C. V., Unchi, S., Binti, M. (2002). Edible insects and entomophagy in Sabah, Malaysia. Malay. Nat. J. 56(2): 131-144.

Dele, R., Conrado, C., Oluwatoyin, B., Oluwole, M. N., Ariana, S., Rafael, M. et al. (2018). Traditional consumption of and rearing edible insects in Africa, Asia and Europe, Critical Crit Rev Food Sci Nutr. DOI: 10.1080/10408398.2018.1440191.

De Foliart, G. R. (2002). The human use of insects as a food resource: a bibliographic account in progress. University of Wisconsin. http://food-insects.com/human-use-insects-food-resource-bibliographic-account-progress/ [Accessed August 15, 2020].

EFSA Scientific Committee. (2015). Risk profile related to production and consumption of insects as food and feed. EFSA j., 13(10): 4257. doi: 10.2903/j.efsa.2015.4257

Fasoranti, J. O., and Ajiboye, D. O. (1993). Some edible insects of Kwara state, Nigeria. Am. Entomol. 39(2): 113-116. doi: 10.1093/ae/39.2.113

Gelfand M. (1971) Insects, pp. 163–171. In Diet and Tradition in African Culture. E&S Livingstone, Edinburgh.

Instar Farming. (2020). Farming crickets for food in the UK. https://www.instarfarming.com/ [Accessed September 1, 2020].

Ghosh, S., Lee, S. M., Jung, C., Meyer-Rochow, V. B. (2017). Nutritional composition of five commercial edible insects in South Kor. J Asia Pac Entomol. 20(2): 686-694. doi: 10.1016/j.aspen.2017.04.003

Halloran, A., Hanboonsong, Y., Roos, N., Bruun, S. (2017). Life cycle assessment of cricket farming in north-eastern Thailand. J. Clean. Prod. 156: 83-94. doi: 10.1016/j.jclepro.2017.04.017

Hanboonsong, Y., and Durst, P. B. (2014). Edible insects in Lao PDR: building on tradition to enhance food security. Food and Agriculture Organization of the United Nations, Bangkok, Thailand. 55.

Hanboonsong, Y., Jamjanya, T., Durst, P. B. (2013). Six-legged livestock: edible insect farming, collection and marketing in Thailand. RAP publication, 3. Regional Office for Asia and the Pacific of the Food and Agriculture Organization of the United Nations, Bangkok.

Hanboonsong Y. (2010). Edible insects and associated food habits in Thailand. *In* Durst P.B., Johnson D.V., Leslie R.N. and Shono K. (eds.) *Forest insects as food: humans bite back Proceedings of a workshop on Asia-Pacific resources and their potential for development*, pp. 173-182. FAO, Bangkok.

Hanboonsong Y., Rattanapan A., Utsunomiya Y. and Masumoto K. (2000) Edible insects and insect-eating habits in Northeast Thailand. *Elytra* 28(2): 355-364.

Harris, W. V. (1940). Some notes on insects as food. Tanganyika Notes and Records. 9: 45-48.

Hoare, A. L. (2007). The use of non-timber forest products in the Congo Basin: Constraints and Opportunities. New York: Rainforest Foundation. Available at: http://tinyurl.com/oyqohag [Accessed: August 20, 2020]

Hwang, B. B., Chang, M. H., Lee, J. H., Heo, W., Kim, J. K., Pan, J. H., et al. (2019). The edible insect *Gryllus bimaculatus* protects against gut-derived inflammatory responses and liver damage in mice after acute alcohol exposure. Nutrients, 11(4): 857. doi: 10.3390/nu11040857

Jongema, Y. (2017). List of edible insects of the world. Laboratory of Entomology, Wageningen University: Wageningen, The Netherlands.

Jongjaithet, N., Wacharangkoon, P., Paomueng Prapasiri, P. (2008). Online. Protein quality and fat content in common edible insects. Division of Nutrition, Ministry of Public Health. (MOPH). Available at: http://nutrition.anamai.moph.go.th/temp/main/view.php?group=3&id=120/ [Accessed September 20, 2020).

Józefiak, D., Józefiak, A., Kierończyk, B., Rawski, M., Świątkiewicz, S., Długosz, J., Engberg, R. M. (2016). 1. a review. Ann. Anim. Sci. 16(2): 297-313. doi: 10.1515/aoas-2016-0010

Kelemu, S., Niassy, S., Torto, B., Fiaboe, K., Affognon, H., Tonnang, H., et al. (2015). African edible insects for food and feed: inventory, diversity, commonalities and contribution to food security. JIFF. 1(2): 103-119. doi: 10.3920/JIFF2014.0016

Lumsa-ad, C. (2001). Study on the species and the nutrition values of edible insects in upper southern Thailand. Kaen Kaset, 29(1): 45-49.

Magara, H. J., Tanga, C. M., Ayieko, M. A., Hugel, S., Mohamed, S. A., Khamis, F. M., et al. (2019). Performance of newly described native edible cricket *Scapsipedus icipe* (Orthoptera: Gryllidae) on various diets of relevance for farming. J. Econ. Entomol. 112(2), 653-664. doi: 10.1093/jee/toy397

Magara, J.O.H., Tanga, C.M., Ayieko, M. A., Hugel, S., Coopeland, R.S., Samira, A. M., et al. (2018). Effect of rearing substrates on the fitness parameters of newly recorded edible cricket

Scapsipedus marginatus in Kenya".Volume 4(supplementary 1) JIFF. DOI: 10.3920/JIFF2018.S1

Mbata, K. J., Chidumayo, E. N., Lwatula, C. M. (2002). Traditional regulation of edible caterpillar exploitation in the Kopa area of Mpika district in northern Zambia. J. Insect Conserv. 6(2): 115-130. doi: 10.1023/A:1020953030648

Mbata, K. J. (1995). Traditional use of arthropods in Zambia. I. the food insects. Food Insects Newsletters. 8(1), 5-7.

Megido, R.C., Alabi, T., Nieus, C., Blecker, C., Danthine, S., Bogaert, J., et al. (2016). Optimisation of a cheap and residential small-scale production of edible crickets with local by-products as an alternative protein-rich human food source in Ratanakiri Province, Cambodia. J Sci Food Agric 96: 627–632. doi.org/10.1002/jsfa.7133

Miech, P., Berggren, Å., Lindberg, J. E., Chhay, T., Khieu, B., Jansson, A. (2016). Growth and survival of reared Cambodian field crickets (*Teleogryllus testaceus*) fed weeds, agricultural and food industry by-products. JIFF. 2(4): 285-292. doi: 10.3920/JIFF2016.0028

Mlček, J., Adámková, A., Adámek, M., Borkovcová, M., Bednářová, M., Kouřimská, L. (2018). Selected nutritional values of field cricket (*Gryllus assimilis*) and its possible use as a human food. Indian J Tradit Know. 17(3): 518-524

Nkouka, E. (1987). Les insectes comestibles dans lês societes d'Afrique Centrale. Muntu: revue scientifique et culturelle du CICIBA, (6), 171-178.

Oibiokpa, F.I., Akanya, H.O., Jigam, A.A., Saidu, A.N. (2017). Nutrient and Antinutrient Compositions of Some Edible Insect Species in Northern Nigeria. Fountain IJONAS. 6(1): 9-24.

Orinda, M. A. (2018). Effects of Housing and Feed on Growth and Technical Efficiency of Production of *Acheta domesticus* (L) AND *Gryllus bimaculatus* for Sustainable Commercial Crickets Production in The Lake Victoria Region, Kenya (Doctoral dissertation, JOOST). Available at: http://ir.jooust.ac.ke:8080/xmlui/handle/123456789/8852 [Accessed on September 25, 2020.

Otieno, M. H., Ayieko, M. A., Niassy, S., Salifu, D., Abdelmutalab, A. G., Fathiya, K. M., et al.(2019). Integrating temperature-dependent life table data into Insect Life Cycle Model for predicting the potential distribution of *Scapsipedus icipe* Hugel & Tanga. PloS one, 14(9): e0222941. doi: 10.1371/journal.pone.0222941

Raksakantong, P., Meeso, N., Kubola, J., Siriamornpun, S. (2010). Fatty acids and proximate composition of eight Thai edible terricolous insects. Food Res Int. 43(1): 350–355. doi:10.1016/j.foodres.2009.10.014

Ramos-Elorduy, J. (2009). Anthropo-entomophagy: Cultures, evolution and sustainability. Entomol. Res. 39(5), 271-288. doi: 10.1111/j.1748-5967.2009.00238.x

Riggi, L., Veronesi, M., Verspoor, R., MacFarlane, C., Tchibozo, S. (2013). Exploring Entomophagy in Northern Benin-Practices, Perceptions and Possibilities. Benin Bugs Report. Bugsforlife, London, UK.

Schmitschek, E. (1968). Insekten als Nahrung, in Brauchtum, Kult und Kultur. Handbuch der Zoologie-eine Naturgeschichte der Stämme des Tierreichs. In: Helmcke, J.G., Stark, D., Wermuth, H. (eds) Handbuch der Zoologie- eine Naturgeschichte der Sta¨mme des Tierreichs, Band 4. Akademie Verlag, Berlin Bd, 3, 1-62.

Singh, O. T., Nabam, S., Chakravorty, J. (2007). Edible insects of Nishi tribe of Arunachal Pradesh. Hexapoda, 14(1), 56-60.

Ssepuuya, G., Sengendo, F., Ndagire C., Karungi, J., Fiaboe K.K.M., Efitre et al. (2020). Effect of alternative rearing substrates and temperature on growth and development of the cricket Modicogryllus conspersus (Schaum). JIFF. ARTICLE IN PRESS.

Stein, C., Florence, D., Yacouba, K., Kariba, C., Stefan, J. (2013). Potential approach to regulate and monitor moisture for *Brachytrupes membreneus* eggs for cricket rearing in the village of sanambele, Mali Poster. pp 1.

Tanga, C., Magara, H. J., Ayieko, A. M., Copeland, R. S., Khamis, F. M., Mohamed, S. A., et al. (2018). A new edible cricket species from Africa of the genus *Scapsipedus*. Zootaxa, 4486(3): 383-392. doi: 10.11646/zootaxa.4486.3.9

Tang, C., Yang, D., Liao, H., Sun, H., Liu, C., Wei, L., Li, F. (2019). Edible insects as a food source: a review. Food Production, Processing and Nutrition, 1(1): 8. doi: 10.1186/s43014-019-0008-1

Tchibozo, S., Van Huis, A., Paoletti, M.G. (2005). Notes on edible insects of South Benin: a source of protein. In: Paoletti, M.G. (ed.) Ecological implications of minilivestock: role of rodents, frogs, snails, and insects for sustainable development. Science Publishers, Enfield, MT, USA, 245-251.

Van der Meer Mohr, J. V. D. (1965). Insects eaten by the Karo-Batak people (a contribution to entomo-bromatology). Entomologische Berichten. Amsterdam. 25: 101-107.

Van Huis, A., Van Itterbeeck, J., Klunder, H., Mertens, E., Halloran, A., Muir, G., et al. (2013). Edible insects: future prospects for food and feed security (No. 171). Food and Agriculture Organization of the United Nations. 201.

Van Itterbeeck, J., Rakotomalala Andrianavalona, I. N., Rajemison, F. I., Rakotondrasoa, J. F., Ralantoarinaivo, V. R., Hugel, S., et al. (2019). Diversity and use of edible grasshoppers, locusts, crickets, and katydids (Orthoptera) in Madagascar. Foods. 8(12): 666. doi: 10.3390/foods8120666

Wang, D., Zhai, S. W., Zhang, C. X., Bai, Y. Y., An, S. H., Xu, Y. N. (2005). Evaluation on nutritional value of field crickets as a poultry feedstuff. Asian-Australas J. Anim. Sci. 18(5): 667-670. doi: 10.5713/ajas.2005.667

Weiping, Y., Junna, L., Huaqing, L., Biyu, Lv. (2017). Nutritional Value, Food Ingredients, Chemical and Species Composition of Edible Insects in China. Web of Science™ Core Collection (BKCI). Pp1-29. http://dx.doi.org/10.5772/intechopen.70085.

Weaving, A. (1973). Insects: A Review of Insect Life in Rhodesia. Regal Publishers. Irwin Press Ltd., Salisbury.

Yhoung-Aree, J. and Viwatpanich, K. (2005) ‘Edible insects in the Lao PDR, Myanmar, Thailand and Vietnam’, in M.G. Paoletti (ed.), Ecological Implications of Minilivestock: Potential of Insects, Rodents, Frogs and Snails, pp. 415–40, Enfield, NH: Science Publisher, Inc.

Zielińska, E., Karaś, M., Jakubczyk, A. (2017). Antioxidant activity of predigested protein obtained from a range of farmed edible insects IJST. 52(2): 306-312. doi: 10.1111/ijfs.13282
